# Notch-dependent epithelial fold determines boundary formation between developmental fields in the *Drosophila* antenna

**DOI:** 10.1371/journal.pgen.1006898

**Published:** 2017-07-14

**Authors:** Hui-Yu Ku, Y. Henry Sun

**Affiliations:** 1 Institute of Genome Sciences, National Yang-Ming University, Taipei, Taiwan; 2 Institute of Molecular Biology, Academia Sinica, Taipei, Taiwan; Harvard Medical School, Howard Hughes Medical Institute, UNITED STATES

## Abstract

Compartment boundary formation plays an important role in development by separating adjacent developmental fields. *Drosophila* imaginal discs have proven valuable for studying the mechanisms of boundary formation. We studied the boundary separating the proximal A1 segment and the distal segments, defined respectively by Lim1 and Dll expression in the eye-antenna disc. Sharp segregation of the Lim1 and Dll expression domains precedes activation of Notch at the Dll/Lim1 interface. By repressing *bantam* miRNA and elevating the actin regulator Enable, Notch signaling then induces actomyosin-dependent apical constriction and epithelial fold. Disruption of Notch signaling or the actomyosin network reduces apical constriction and epithelial fold, so that Dll and Lim1 cells become intermingled. Our results demonstrate a new mechanism of boundary formation by actomyosin-dependent tissue folding, which provides a physical barrier to prevent mixing of cells from adjacent developmental fields.

## Author summary

During development, boundary formation between adjacent developmental fields is important to maintain the integrity of complex organs and tissues. We examined how boundaries become established between adjacent developmental fields—which are defined by expression of distinct selector genes and developmental fates—using the *Drosophila* eye-antennal disc as a model. We show that boundary formation is a progressive process. We focused our analysis on the antennal A1 fold that separates the A1 and A2-Ar segments, corresponding to the evolutionarily conserved segregation between coxopodite and telopodite segments of arthropod appendages. We describe a clear temporal and causal sequence of events from selector gene expression to establishment of a lineage-restricting boundary. We found that Notch activation at the boundary between adjacent fields of selector gene expression triggers actomyosin-mediated cell apical constriction, which induces the formation of an epithelial fold and prevents intermixing of cells from adjacent fields. Our findings describe a novel mechanism by which epithelial fold provides a physical barrier for cell segregation.

## Introduction

During development, an organism is progressively divided into discrete fields that develop into different organs or parts of an organ. In many cases, the adjacent developmental fields develop distinct morphological, functional and molecular characteristics and are often divided by a sharp boundary that function to prevent lineage-related cells originating from one compartment from crossing into the adjacent compartment. Such lineage-restricting boundaries were first described in the fruitfly *Drosophila* wing and the milkweed bug *Oncopeltus* abdomen, using mitotic clones and cuticle markers to trace lineage distributions [1, 2]. The same phenomenon was then reported for other parts of the fly body and in vertebrates [3–10]. Nevertheless, not all boundaries have been analyzed for lineage restriction at single cell resolution.

Compartment boundaries generally coincide with the expression borders of the selector genes that determine the fates of developmental fields. For example, in the fly wing disc, the anterior-posterior (A/P) boundary correlates with the border of *engrailed* (*en*) expression in the posterior compartment, whereas the dorsal-ventral (D/V) boundary correlates with the border of *apterous* (*ap*) expression in the dorsal compartment. The expression domain of the selector genes does not begin as a sharply defined pattern (*e*.*g*. [11]), and usually evolves from a weak and fuzzy to a strong and sharply defined pattern through positive and negative regulation with other genes. Mutual repression between two selector genes, either direct or indirect, can force a cell at the expression border to express only one of the two selector genes. However, the cell-autonomous cell fate may result in a rough border of two cell types. A smooth and sharp alignment may require additional mechanisms to coordinate the cells at the expression border. Hence, the expression border and the lineage-restricting boundary are two phenomena characterized by different, though coinciding, processes. Therefore, the relationship between gene expression borders and lineage-restricting boundaries needs to be considered with respect to their temporal progression. We define ‘boundary’ as indicating lineage restriction, ‘compartment boundary’ to indicate absolute lineage restriction, ‘field boundary’ for incomplete lineage restriction, and ‘border’ to refer to expression domains.

Three types of mechanisms have been shown to play a role in boundary formation and maintenance. First, differential cell affinities modulated by cadherin interactions are responsible for various boundary formations [12–15]. Second, reduced cell proliferation found at the vertebrate somite and *Drosophila* D/V boundary can minimize movements resulting from mitosis [16–18]. However, whether reduced cell proliferation or bias in mitosis orientation is important for the maintenance of the boundary is unclear [11, 19, 20]. Third, mechanical forces provided by the intracellular cytoskeletal network can sharpen boundaries in both the vertebrate and invertebrate system [11, 19, 21–30]. For instance, actomyosin cables are responsible for cell partitioning in *Drosophila* A/P and D/V boundaries, as well as zebrafish rhombomeric boundaries [19, 21, 25, 26]. Actomyosin cables bind to adherens junctions to form belt-like supracellular structures [31, 32]. These cables are enriched for cells along the boundary, serving as physical barriers that restrict cells in adjacent compartments from mixing, with or without morphological changes [19, 25–27].

**Fig 1:**
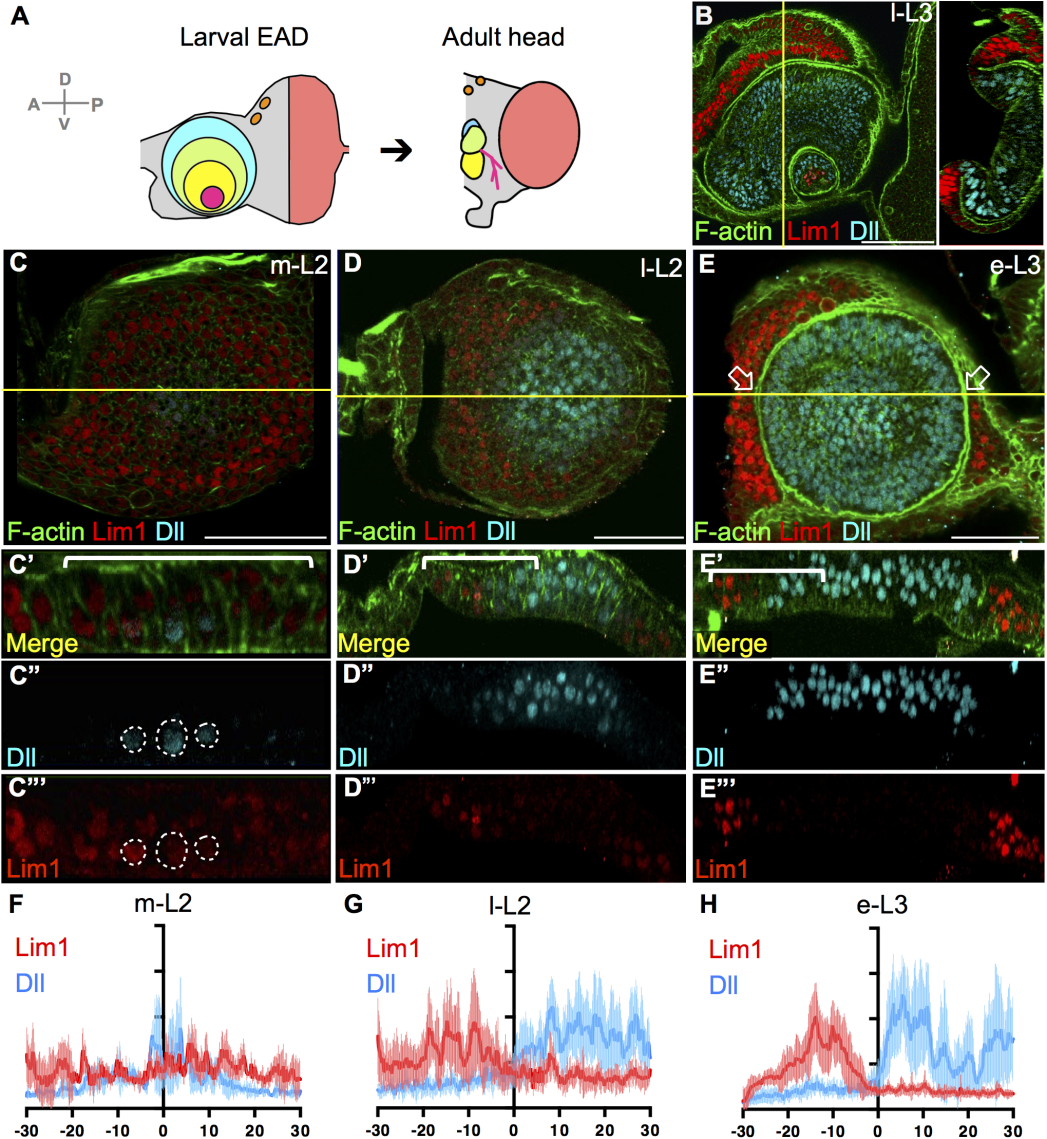
Progressive sharpening of selector gene expression and the formation of epithelial folds in the EAD. (A) Correspondence of developmental fields between late third instar (l-L3) EAD and the adult head. Red: eye; blue, green, yellow, and pink: proximal to distal (A1, A2, A3, and Ar) antennal segments; orange: ocelli; grey: head cuticle. (B) Lim1 (red) and Dll (blue) expressions are separated in the l-L3 EAD by an epithelial fold (F-actin, green). (C-E) *w*^*1118*^ EAD analyzed for morphological changes (F-actin, green), and gene expression patterns (Lim1, red; Dll, blue) in m-L2 (26-32h AEH, C-C”‘), l-L2 (38-44h AEH, D-D”‘) and e-L3 (48h AEH, E-E”‘). Z-axis projections at the yellow lines show the epithelial morphology in lateral view. (C) Dashed lines indicate m-L2 cells coexpressing Lim1 and Dll. (E) Arrows point to the fold at the Lim1/Dll expression border. (F-H) Dll (blue) and Lim1 (red) expression levels—based on pixel intensities (Y axis) and individually normalized against background—were quantified from the bracketed region (X axis, total 60 μm) in C’, D’ and E’. The center (0 in the X axis) was manually positioned at the fold (e-L3) or at the Dll-Lim1 overlapping regions (L2). All images in this and subsequent figures are oriented as dorsal-face up and posterior end to the right, with cross-sections oriented with the apical surface of the disc proper to the right or top. Scale bars: 50μm.

We used the larval eye-antenna disc (EAD) to explore the mechanism of boundary formation in the *Drosophila* head, with an emphasis on the boundaries in the proximal-distal (P/D) axis, *i*.*e*. the boundary between the antennal segments. The EAD is a sac-like tissue composed of monolayered epithelial cells covered by peripodial cells. It contributes to the majority of the adult head organs, including compound eyes, antennae, ocelli, maxillary palps and the head cuticle (Fig 1A). These organs abut each other, with smooth and clear boundaries. The antenna is further divided into six segments, A1-A5 and the most distal arista (Ar). Patterning of P/D antennal segments by critical transcription factors is achieved by *hedgehog* (*hh*)-dependent *decapentaplegic* (*dpp*, in dorsal) and *wingless* (*wg*, in ventral) inductions [33]. In the center and marginal antennal disc, which are destined to be the distal and proximal antennal segments, respectively, *Distal-less* (*Dll*) and *homothorax* (*hth*) are activated upon high and low levels of Dpp and Wg [33–36]. Cells that coexpress *hth* and *Dll* become the A2 to A4 segments [37]. The LIM-homeodomain protein Lim1, which is regulated by EGFR signaling, specifies the A1 and Ar segments [38–40].

Here, by examining the temporal sequence of Dll and Lim1 gene expressions, lineage restriction, and tissue morphogenesis, we report that the boundary separating the most proximal segment (Lim1-expressing, A1), from the more distal parts (Dll-expressing) of the antenna involves a Notch-dependent downregulation of *bantam* microRNA and de-repression of Enable (Ena). Strikingly, this pathway produces an epithelial fold that not only acts as a boundary to ensure cells stay within their respective fields, but also reinforces Notch signaling, thereby safeguarding boundary integrity. Thus, our results have uncovered a novel mechanism for the establishment of a field boundary that involves the formation of folded epithelial structures.

## Results

### Progressive sharpening of gene expressions and the formation of epithelial folds in the EAD

The EAD undergoes a series of progressive epithelial folds from the early third instar stage (e-L3, S1A Fig). EAD cells are cuboidal in the early second instar stage (e-L2). From the late second instar (l-L2) (S1B Fig), epithelial cells in the antennal and eye fields become columnar, but medial cells remain cuboidal and have a concave morphology in lateral view (S1B and S1B’ Fig). During e-L3, a ring fold (hereafter termed the ‘A1 fold’) is formed to separate the prospective A1 antennal segment from the distal A2-Ar antennal segments (S1C Fig). Also during e-L3, an E/C fold that separates the eye and head cuticle partially extends from the lateral to medial regions (S1C–S1C Fig), becoming complete by the late third instar (l-L3) (S1D–S1D” Fig). A fold that separates the most distal arista segment (Ar, termed the ‘Ar fold’ hereafter) and the other antennal segments forms during l-L3 (S1D Fig). In the l-L3 antennal disc, the A1 fold correlates with the border separating the Dll and Lim1 expression domains (Fig 1B). Dll is expressed in the A2-Ar segments, whereas Lim1 is specifically expressed in the A1 segment and the head cuticle. In the mid second instar (m-L2) EAD (Fig 1C, dashed line, 1F), before the A1 fold has been formed, Dll and Lim1 expressions are weak and partially overlap (co-expression), exhibiting a fuzzy border due to two to three rows of cells co-expressing Dll and Lim1. From l-L2 (Fig 1D and 1G) to e-L3 (Fig 1E and 1H), levels of Dll and Lim1 gradually increase and become sharply confined. At e-L3, the border between the Lim1 and Dll expression domains sharpens and the genes are rarely co-expressed (S2A–S2C Fig). The sharp cell-autonomous segregation of Dll and Lim1 expression begins before formation of the A1 fold, suggesting that the epithelial fold is not the cause of segregated the expression.

The distal A2-Ar segments specified by the Dll gene correspond to the evolutionarily conserved telopodite in arthropod appendages. Therefore, the A1 fold separates the proximal coxopodite from the distal telopodite. We hypothesize that the folded tissue architecture at the A1 fold may act as a lineage-restricting boundary between the proximal Lim1-dependent coxopodite and the distal Dll-dependent telopodite.

### Progressive lineage restriction coincides temporally with fold formation

**Fig 2:**
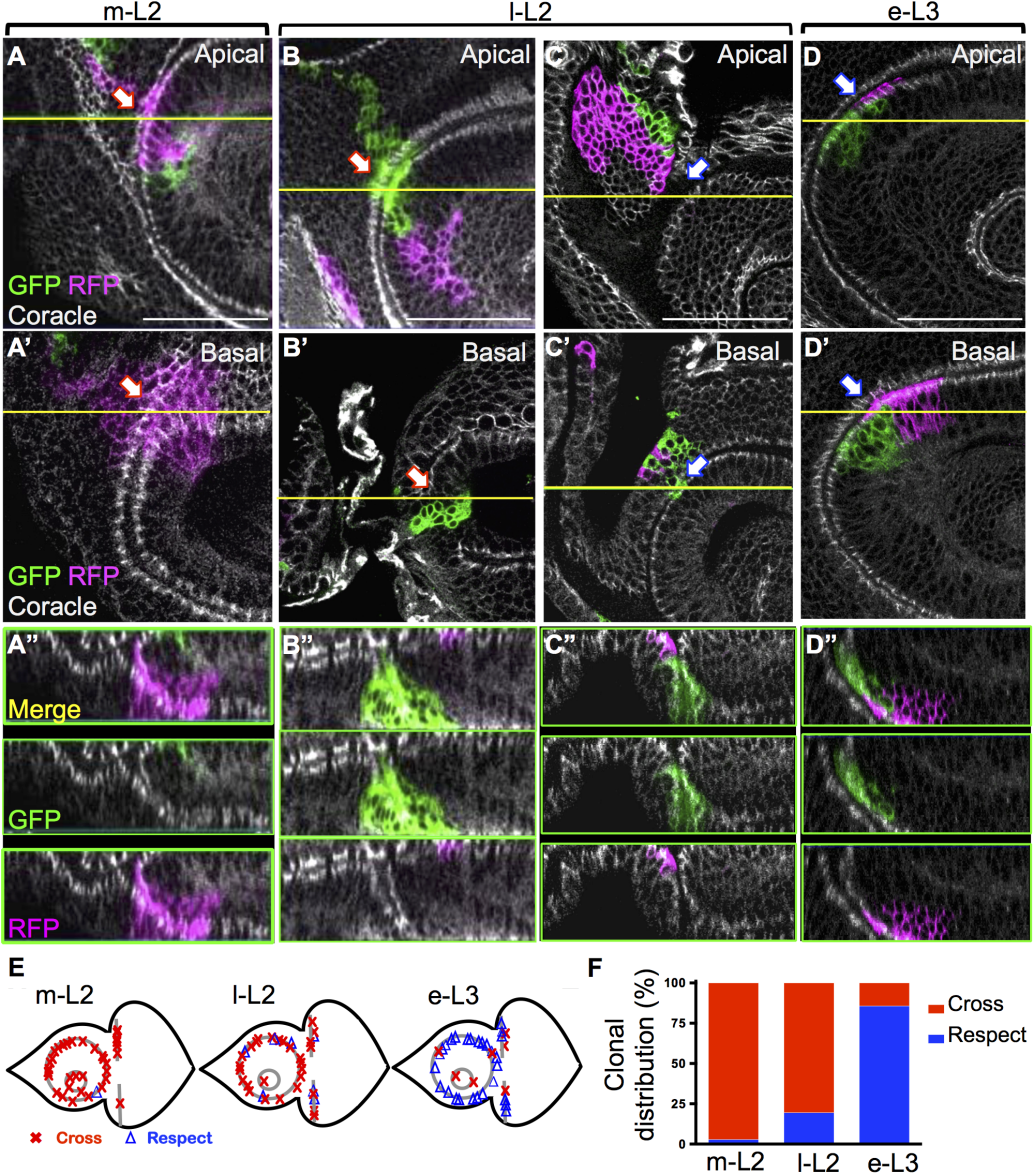
Lineage restriction coincides with fold formation. (A-D) TSM clones were induced at the indicated time and examined in l-L3 EAD. For lineage restriction analysis, only those clones located at or spanning the respective borders were scored. Clones were examined along XZ and YZ optical sections to determine whether the clone crossed the boundary at the bottom of the fold. (A-D) Apical XY planes. (A’, B’, C’ and D’) Basal XY planes. (A”, B”, C” and D”) The Z-axis projection images along the yellow line are shown. Red and blue arrows indicate clone that crossed or were restricted by the boundary, respectively. (E) Summary of the locations of all meaningful clones examined. Clones crossing or restricted by the boundary are indicated by a red cross or blue triangle, respectively. (F) The percentages of clonal patterns at the three stages are plotted; numbers of analyzed clones: m-L2 = 34, l-L2 = 37, e-L3 = 35. Scale bars: 50μm.

Next, we tested whether the A1 fold serves as a lineage-restricting boundary. The classical definition of a compartment boundary in *Drosophila* depends on cuticular markers (*e*.*g*. *yellow* (*y)* and *multiple wing hair* (*mwh)*) for wing, leg and antenna, or pigmentation (*white*, *w*) for compound eye. These markers can only be used on adult tissues. No single marker can be used for both eye and other head structures. We used Twin-Spot MARCM (TSM) to induce sister clones with different fluorescent proteins [41]. The fluorescent markers allowed analysis of clone distribution covering the entire head structure of both larval and adult stages (S3 Fig). Pairing of the sister clones allowed us to determine if a clone was indeed from a single origin. The TSM clones were induced at indicated time-points, and their distributions were analyzed in l-L3 discs (S3C–S3F Fig, and Fig 2). TSM clones in wing and antennal discs determined the timing of A/P and D/V boundary formation (S3C–S3F Fig). For example, in the wing disc, clones induced in L2 cross the D/V boundary (marked by Cut-expressing cells) but those induced at e-L3 do not, indicating that the D/V boundary is formed at e-L3 and not L2 (S3C–S3D Fig). These results are consistent with previous reports, and validate our TSM clonal analysis for the study of lineage restrictions.

We examined the distribution of the TSM clones relative to the A1 fold. All clones at the folds were examined in different focal planes to check whether they crossed or were restricted by the A1 fold. Even clones for which 1–2 cells crossed the A1 fold were counted as having crossed it. Therefore, our clonal analysis is defined by a very sharp border at single cell resolution. When clones were induced at m-L2, all except one of the TSM clones crossed the A1 fold (Fig 2A, red arrow; 2E; 2F, 2.94% restricted by boundary). The frequency of clones that were restricted by the epithelial fold increased when clones were induced at l-L2 (Fig 2B and 2C, blue arrow; 2E; 2F, 18.92% restricted by boundary), and they occurred at the A1 fold and the lateral part of the E/C fold (Fig 2E, blue triangle). Most of the clones induced at e-L3 were restricted by the A1 fold (Fig 2D, blue arrow; 2E; 2F, 85.71% restricted by boundary), and crossed the Ar fold (Fig 2E, red cross; the Ar fold forms in m-L3). The E/C boundary was established progressively, laterally to medially (S1C–S1D Fig, and Fig 2E) because, at e-L3, most lateral clones were restricted by this boundary (6/7), but the medial clones crossed it (2/2).

Our TSM clonal analysis showed that the A1 boundary is not an absolute lineage-restricting boundary. Even if we count clones with a single cell crossing as having been restricted by the A1 fold boundary, the frequency of e-L3 clones respecting the it is less than 100% (88.6% for TSM clones). In contrast, clones induced during the first instar (L1) absolutely respected the A/P boundary in wing disc at a single cell resolution (S3F Fig, marked by Patched, Ptc, 31/31). Since the A1 fold boundary does not fit the classical definition of a compartment boundary, we term it a ‘field boundary’ to differentiate it from a compartment boundary. In summary, lineage restriction at the A1 fold correlates temporally with the formation of the epithelial fold. This supports our hypothesis that the epithelial fold serves as a lineage-restricting boundary.

### Apical constriction and changing cell shape during A1 fold formation

**Fig 3:**
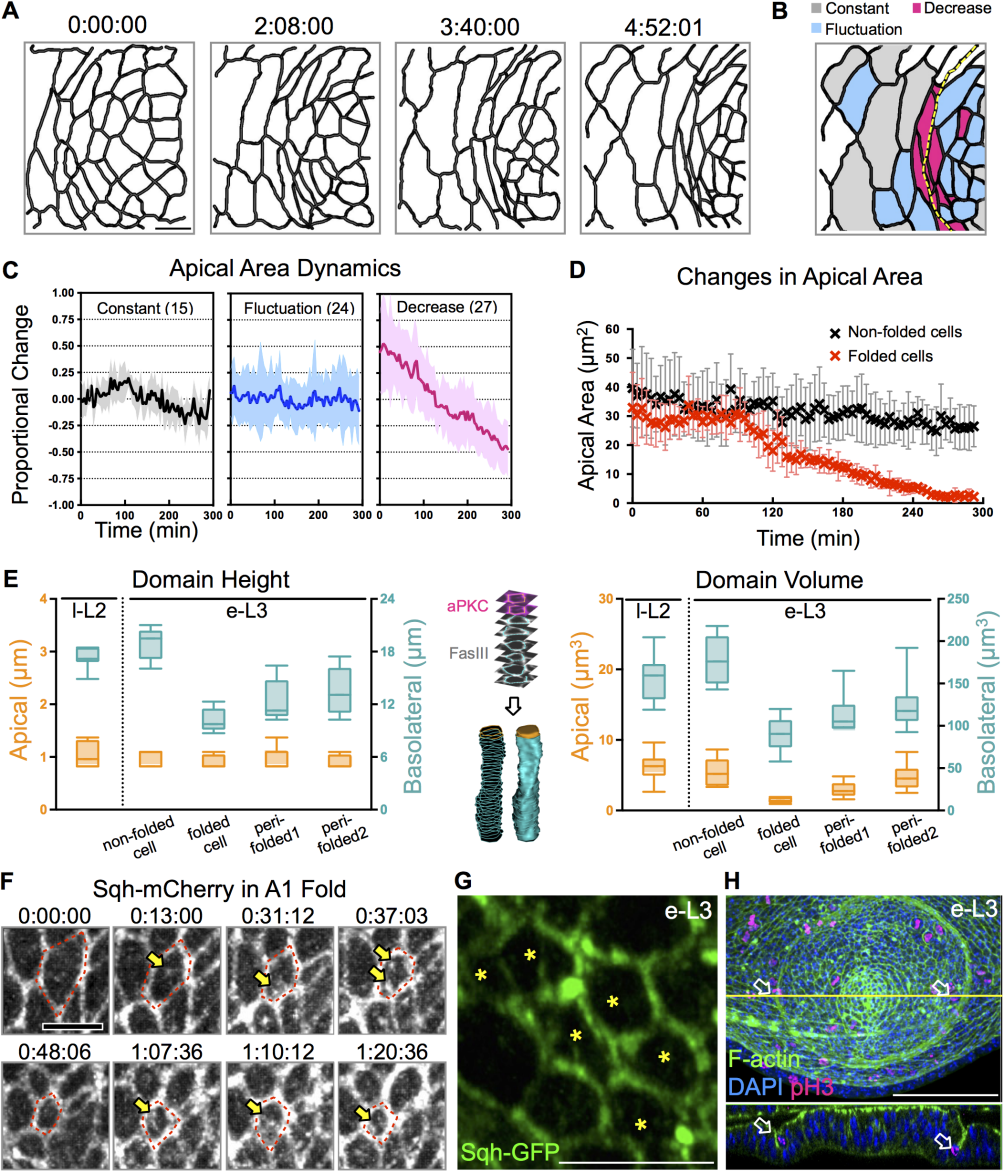
Apical constriction and cell shape changes during A1 fold formation. (A) Time-lapse images of Sqh-GFP in *ex vivo* cultured l-L2 EAD for 5 hours. Imaris filament, and surface-tracing modules were used for individual cell segmentation and tracing. (B) Cells are classified into three groups based on their dynamic changes in the apical area and are shown for T_final_ (4:52:01). Cells that showed a significant reduction in apical size (from 30–40μm^2^ to <10μm^2^) are labeled red. Cells that showed a fluctuating apical area, defined as δArea_i_ (A_maxi_-A_mini_) ≥ 10μm^2^ over time, are labeled blue. Otherwise, cells were considered to have a constant apical area (labeled grey). The yellow dashed line marks the A1 fold at T_final_. (C) Cells in each group are plotted according to by their proportional change in apical area ((A_ti_−A_avgi_) /A_avgi_) over time. Mean ± stdev are shown as a solid line and lightly shaded area, respectively. The ranges of stdev for the constant and the fluctuating groups are 0.05–0.4, and 0.3–0.9, respectively. Analyzed cells in each group are indicated. (D) The apical areas of cells located at the A1 fold were significantly reduced 2h prior to fold formation (at 180min). Mean ± stdev are shown. Cells away from the fold do not show a significant reduction in apical area. (E) Quantitation of cell height and volume from l-L2 and e-L3 EAD. Stack contours from aPKC (apical, magenta) and FasIII (basolateral, white) staining for volume rendering are illustrated in the middle panel. In addition to cells at the A1 fold, cells one row (perifold-1) and two rows (perifold-2) away from the A1 fold were also scored. (F) Sqh-mCherry accumulates periodically in the apical-medial region (yellow arrow) of constricting cells (red dashed line). Original time-lapse images: see S1 Movie. (G) During e-L3, the cells at the fold (marked by stars) exhibit an even distribution of Sqh-GFP (green) in a 2–3μm stack image projection (N = 4). (H) Mitotic cells (pH3, magenta) are observed along the A1 fold (arrow) during e-L3 (DAPI, blue; F-actin, green). EAD presented 3–4 mitotic cells (N = 7). Scale bars: 5μm, except in H for which it is 50μm. Time indicated as hh:mm:ss.

Since the epithelial fold strongly correlated temporally and spatially with the establishment of lineage restriction and the gene expression border, we investigated the process of epithelial fold in the EAD development. Previous studies have shown that apical actomyosin triggers apical constriction to initiate fold [42–44]. Spaghetti-squash (Sqh)—a non-muscle myosin regulatory light chain—is a key component of the actomyosin network [45]. Therefore we examined whether the EAD fold arises from apical constriction by live imaging *ex vivo*-cultured Sqh-GFP from l-L2 EAD (Fig 3A–3C) [46]. Based on their dynamics in the apical area, three groups of cells could be distinguished; namely, constant, fluctuating and decreasing cells (Fig 3B and 3C). Cells exhibiting a significant decrease in apical area coverage over the 5-hour period were located primarily along the A1 fold (Fig 3B and 3C). Fluctuating cells in the apical area were scattered close to the A1 fold (Fig 3B). Cells constant within the apical area were located further away from the A1 fold (Fig 3B and 3C). The extent of EAD apical area reduction is similar to that described for embryonic cells in mesoderm formation (Fig 3D) [42]. Cell height and volume before (l-L2) and after (e-L3) A1 fold formation were measured from fixed EAD for better Z resolution (Fig 3E, details in S1 Table). For cells in the A1 fold, the heights of the apical domains (defined by aPKC) of folded cells were similar to non-folded cells, but the apical volumes were significantly smaller (20% those of non-folded cells), likely due to constriction of the apical area (Fig 3C and 3D). For the basolateral domains (defined by FasIII) of cells in the A1 fold, height and volume were both lower (by 50%) than for non-folded cells, but the difference were not as drastic as for apical volumes and dimensions (Fig 3C–3E). Cells surrounding the A1 fold (*i*.*e*. 1 or 2 rows away from the A1 fold) were slightly taller and larger than folded cells, but these dimensions were still less than those for non-folded cells.

Sqh protein is distributed as junctional and medial-apical species in the EAD. Junctional Sqh was present in all cells, whereas medial-apical Sqh was observed in cells undergoing apical constriction (Fig 3F, arrow, and S1 Movie). Medial-apical Sqh accumulated periodically as apical size decreased (S1 Movie), probably through a mechanism similar to that reported to drive cell invagination during mesoderm formation [42, 43]. In cells at the A1 fold, junctional Sqh was uniformly presented (Fig 3G, marked by stars), unlike the cable-like structure of actomyosin that is enriched at opposing interfaces of cells along the A/P boundary [27]. Mitotic cells were frequently observed in the A1 fold (Fig 3H, arrow), suggesting that lineage restriction at the A1 fold is not likely due to a zone of quiescent cells.

### Myosin activity underlies formation of the epithelial fold to ensure lineage restriction

We next tested whether actomyosin is responsible for the apical constriction and formation of the A1 fold. Actomyosin is composed of actin, non-muscle myosin II heavy chain (Zipper, Zip), and regulatory light chain (Spaghetti-squash, Sqh). Spaghetti-squash activator (Sqa) is a myosin like chain kinase (MLCK)-like kinase required for non-muscle myosin activation [47]. Both *zip*^*2*^ and *sqa*^*f01512*^ mutant clones at the A1 and Ar folds showed reduced fold (S4A–S4C Fig, compare yellow and white arrows), while maintaining apical-basal polarity. Larger mutant clones showed apical swelling and/or delamination, as has been previously reported (S4D Fig) [48].

**Fig 4:**
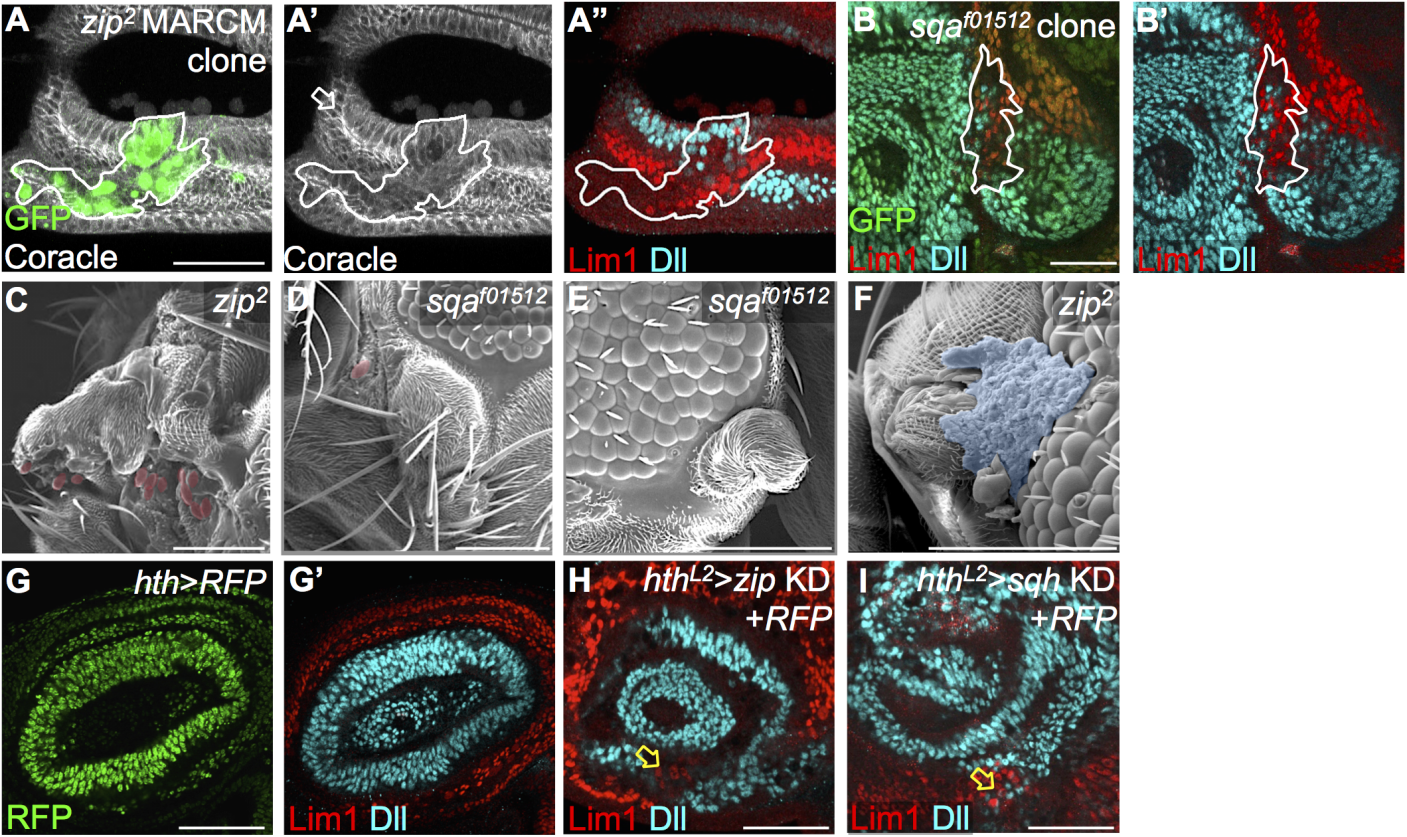
Myosin activity underlies formation of the epithelial fold to ensure lineage restriction. (A-B) *zip*^*2*^ MARCM clone (GFP-positive) and *sqa*^*f01512*^ clone (GFP-negative) show mixing of Lim1 and Dll cells (A” and B’, 18/23 in *zip*^*2*^, 11/15 in *sqa*^*f01512*^, numbers indicates discs with Lim1-Dll mixing/total disc). (A’) Disc morphology is revealed by Coracle (white) staining. White arrow indicates the A1 fold position. (C-E) In adults carrying *zip*^*2*^ or *sqa*^*f01512*^ clones, mislocalized ommatidia (highlighted red in SEM) can be detected in antennal segments (C) or head cuticle (D). (E) Adults with *sqa*^*f01512*^ mutant clones showed antenna-like tissue at the borders of compound eyes. (F) Necrotic-scar like cells were observed in *zip*^*2*^ mutant adults (highlighted in blue). (G-G’) *hth-GAL4* expression (RFP, green) completely covers the Lim1 expression field (red) and partially overlaps with Dll (blue). (H-I) Knockdown of *zip* or *sqh* by *hth-GAL4* from L2 (*hth*^*L2*^: *hth-GAL4+tub-GAL80*^*ts*^, shifted to non-permissive temperature from L2) showed high penetrance of Lim1 and Dll cell mixing (yellow arrow, *zip*: 24/24; *sqh*: 21/22). Scale bars: 50μm, except C-F: 100μm.

We then examined if lineage restriction is affected when epithelial fold is disrupted. Lim1- and Dll-expressing cells are well segregated in the L3 antenna (Fig 1E). In *zip*^*2*^ MARCM and *sqa*^*f01512*^ clones that span the A1 fold, mixing of Dll- and Lim1- expressing cells was observed within the clones (Fig 4A and 4B, 18/23 in *zip*^*2*^ and 11/15 in *sqa*^*f01512*^) and occasionally outside of the clones (S4E–S4E’ Fig, arrow), implying a breakdown of the boundary. *zip*^*2*^ or *sqa*^*f01512*^ clones located exclusively within the A1 or A2-Ar domains did not show altered expression of Lim1 or Dll (S4F Fig), suggesting that cell mixing in these mutant clones was not due to altered cell fates, but to loss of positional restriction. The mislocalized cells were maintained in the epithelial sheet and were not sorted out basally for elimination (S4G Fig). Cleaved caspase 3 in the mutant larval EAD was rarely detected (S4H Fig). Together, these finding imply that the cell mixing phenotype may be observed in adults. It was difficult to observe cell mixing between antennal segments in the adult head. However, adult heads with *zip*^*2*^ or *sqa*^*f01512*^ clones consistently showed mislocalized ommatidia in head cuticle and antennae (Fig 4C and 4D, highlighted in red), and antennal-like tissue at the borders of compound eyes (Fig 4E), indicating a breakdown of the E/C boundary, which is also characterized by an epithelial fold (Fig 2E). We occasionally observed necrotic scar-like cells in *zip*^*2*^ or *sqa*^*f01512*^ adults (Fig 4F), suggesting that some elimination of mutant cells takes place during or after the pupal stage. Knocking down (KD) of *zip* and *sqh* by *hth-GAL4* from the L2 stage, which covers the A1-A3 region in the antennal disc (Fig 4G) [36], revealed disorganization and mixing of Lim1 and Dll cells (Fig 4H and 4I, frequency of cell mixing in *zip* KD: 100%; *sqh* KD: 95.5%). Again, these mislocalized cells were properly integrated in the epithelial sheet for both *zip* and *sqh* knockdown (S4I–S4J Fig). We observed Dll cells in the Lim1 field (Fig 4I and S4G–S4I Fig) and Lim1 cells in the Dll field (Fig 4A, 4B, 4H and S4J Fig), so mislocalization between different fields due to disruption of the A1 fold is reciprocal. Collectively, these results support a role for the epithelial fold in acting as a boundary to separate different cells in the EAD.

We also tested a number of proteins known to interact with actomyosin and that are involved in boundary formation to clarify their roles in the A1 fold formation. Knockdown by *hth-GAL4* of the basal focal adhesion components integrin (encoded by *myospheroid*, *mys*) and talin (encoded by *rhea*) (S5A–S5D Fig), the Hippo-regulating LIM protein Ajuba (*jub*) (S5E–S5F Fig) [49], and the adherens junction component Echinoid (*ed*) [50] (S5G–S5H Fig) did not affect formation of the A1 fold or segregation of Lim1 and Dll cells. These results show that integrin, talin, Ajuba, and Ed are not likely to be involved in A1 boundary formation. However, these mutant cells showed various morphological defects (*e*.*g*. swelling, enlargement or delamination) to a similar extent as *zip*^*2*^ cells (compare S4D Fig to S5I–S5L Fig; quantitation in S5M Fig). Our results imply that drastic changes in cell shape *per se* do not affect cell segregation at fold-mediated boundaries. Therefore, the mixing of Dll and Lim1 cells in *zip*, *sqh*, and *sqa* mutants is not due to altered cell size or morphology, but due to disruption of the epithelial fold.

### Disruption of myosin activity using CALI permits boundary crossing

**Fig 5:**
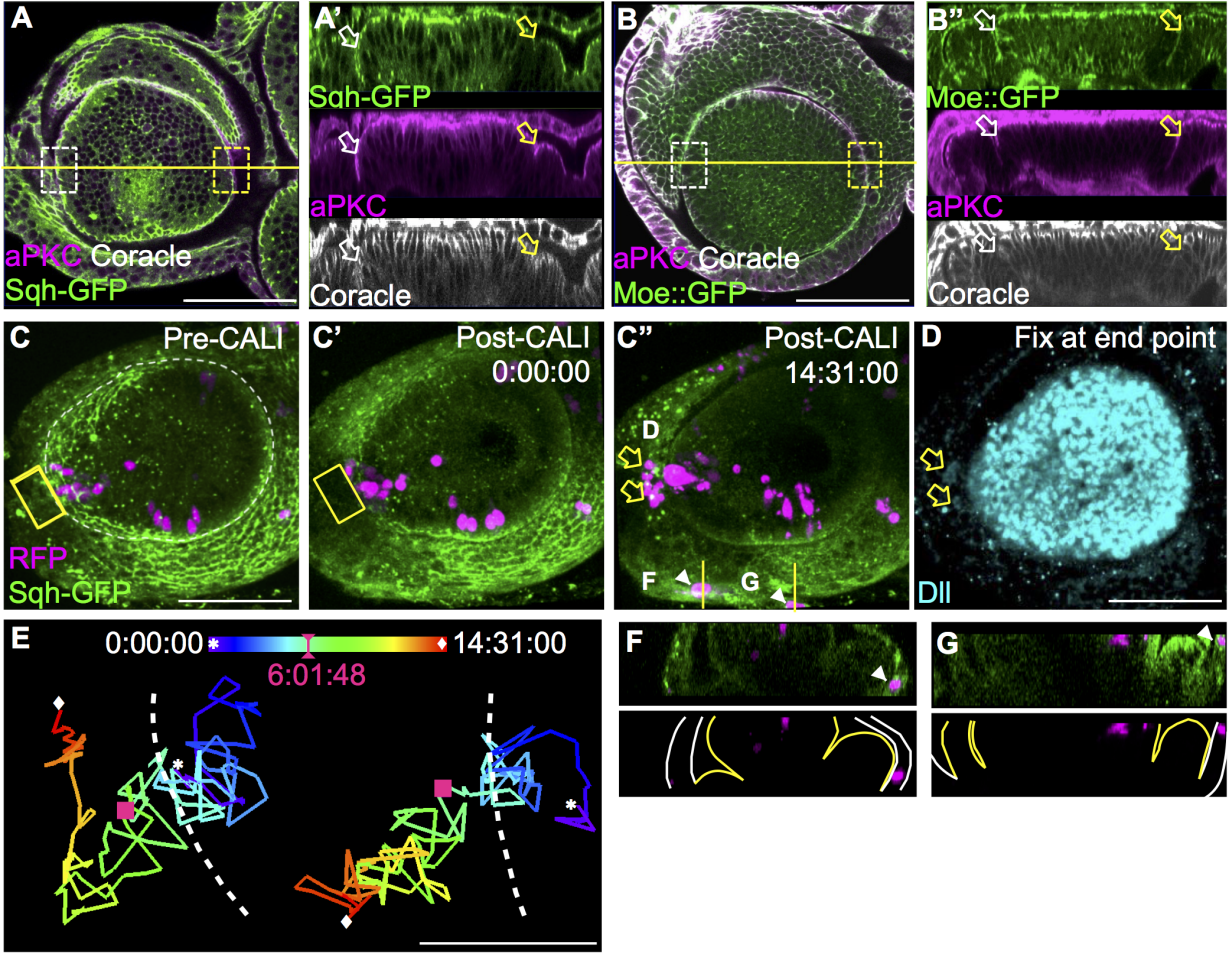
Disruption of myosin activity using CALI permits boundary crossing. (A) Inactivation of actomyosin by Sqh-GFP-mediated CALI. *Ex vivo*-cultured e-L3 EAD were fixed immediately after CALI treatment (see Materials and Methods). EAD morphology is revealed by Sqh-GFP (green), aPKC (magenta) and Coracle (white) staining. The fold in the control region (non-CALI, white box) and CALI treated region (yellow box) were compared. (A’) In contrast to the control (non-CALI, white arrow), the CALI-treated region (yellow arrow) exhibits a significant reduction in the extent of tissue fold. (B) The actin-binding ERM protein Moe was used as a control for CALI specificity. Expression of Moe::GFP (green) only decorates actin filaments without notable effects. (B’) CALI on Moe::GFP does not affect the A1 fold (compare the non-CALI, white box/arrow, and the CALI-treated region, yellow box/arrow). (C-D) CALI in combination with a clonal tracing experiment. Cells expressing RFP (magenta) were induced upon heat-shock in L2. After 24h, the EAD was dissected, treated with CALI, and monitored for 14-16h. The images were shown in 3D projection. (C-C”) In cultured EAD, inactivation of Sqh-GFP (green) via CALI (yellow boxed region) at the A1 fold (white dashed line) showed that the RFP cells originated from the Dll field across the A1 boundary (arrows in C”, see S2 Movie) and maintained Dll expression (D, blue). (D) Dll expression (blue) after live imaging for 14h. N = 3. (E) Trajectory of the two RFP cells that cross the A1 fold (as cells pointed by yellow arrows in C”). Red squares indicate the RFP positions at post CALI time 6:01:48, with overall trajectory shown in color-coded time map (dashed line: A1 fold). Star and diamond indicate position of RFP cells at T_0_ and T_final_ post CALI treatment, respectively. (F-G) Cross sections to show that the two RFP clones in C”(marked by white arrowheads) that seemed to have crossed the A1 boundary are actually located in the peripodial epithelium. White and yellow lines outline peripodial and disc proper, respectively. Scale bars: 50μm, except in E: 10μm. Time indicated as hh:mm:ss.

To assess the effect of acute blockage of Sqh on the formation of the A1 boundary, we used chromophore-assisted laser inactivation (CALI) [27, 51, 52] to specifically inactivate Sqh-GFP in *ex vivo* e-L3 EAD. Indeed, Sqh-GFP inactivation by CALI caused a significant reduction in the extent of epithelial fold in the A1 fold (Fig 5A–5A’, compare aPKC and Coracle signals in the boxed regions for CALI and control). In contrast, the same CALI treatment on Moe-ABD::GFP did not cause a similar effect (Fig 5B–5B’), indicating the high specificity of CALI [27, 53]. Clones expressing RFP were induced at L2. Cells adjacent to, but not including RFP-labeled clones, were subjected to CALI treatment (S2 Movie). When CALI was applied to the A1 fold (Fig 5C, yellow boxed region), a few cells from an adjacent RFP clone crossed the disrupted A1 fold to the adjacent field (S2 Movie). Cells that crossed the A1 fold still maintained their Dll expression (yellow arrow in Fig 5C and 5D), indicating that cell fate had not changed (at least for the time span of our observations).

Due to the EAD curvature, some RFP-labeled cells from the peripodial membrane appeared in several time points that may confuse the observation (white arrow head in Fig 5C”; cross section in 5F and 5G,). Individual cell tracking was performed over time to unambiguously show border crossing (S2 Movie, overall trajectory in Fig 5E). The CALI inactivation of Sqh-GFP only lasted less than 5–6 hours, after which the endogenous Sqh-GFP expression was recovered and the A1 fold was reformed (S6A Fig, arrow). Hence the time window for RFP cell across boundary was less than the first 6 hours post CALI treatment (Fig 5E, S2 Movie). In the EAD *ex vivo* culture, we also noticed some small nuclei appeared at later time points (from post CALI 10h), probably result from impaired growth and gross morphological changes under *ex vivo* condition [46]. Indeed, the EAD cultured for more than 12 hours showed notable cellular architectural and morphological alterations, which was not observed at 6h post-CALI time point (compare S6A–S6B Fig). Therefore, the EAD deterioration after long-term culture is unlikely to contribute to border crossing. RFP clones in the EAD without CALI treatment was unable to cross the A1 fold (S3 Movie). We also analyzed the trajectory of RFP clones that crossed (CALI) or not crossed (non-CALI) the A1 fold (S6C Fig). The orientation and displacement are comparable between crossed and not crossed cells, indicating that CALI treatment did not cause significant side effects and further lead to additional behavior changes.

### Notch activation drives folding of the A1 boundary

**Fig 6:**
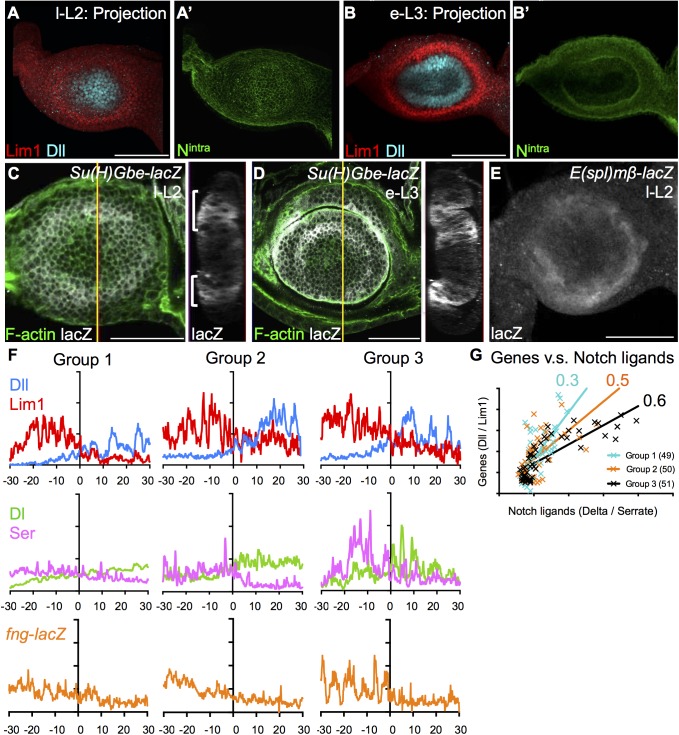
Notch activation and differential expressions of Delta, Serrate and *fng*-*lacZ* precede epithelial fold. (A-E) N activation is indicated by N^intra^, (A’, B’, green, shown in 3D projection) and the N transcriptional reporters *Su(H)Gbe*-*lacZ* (C, D, white) and *E(spl)mβ* -*lacZ* (E, white) in an antennal disc from l-L2 (A,C, E) to e-L3 (B,D). N^intra^ is low and relatively uniform in l-L2 (A) and highly enriched at the Lim1/Dll expression border in e-L3 (B). (C, D) *Su(H)Gbe*-*lacZ* shows a circular pattern before (C, l-L2) and after (D, e-L3) A1 fold. In the cross section, as shown on the right for C and D, the reporter intensity is strongest at the center of the putative fold and gradually declines in surrounding cells (marked by brackets). (E) *E(spl)mβ-lacZ* expression also appears in a circular pattern, corresponding to the future A1 fold at l-L2. (F) Expression of the selector genes Lim1 (red) and Dll (blue), the N ligands Dl (green) and Ser (magenta), as well as *fng*-*lacZ* (orange) were analyzed prior to tissue fold in three groups of discs of increasing size putatively representing increasing developmental time. Pixel profiling shows clear segregation of Dll/Lim1 expression in groups 1, 2 and 3. Segregation of Dl/Ser expression only begins in group 2 and becomes clear in group 3. Dl is high in the central (Dll-expressing) region, and Ser is high in the peripheral (Lim1-expressing) region. *fng*-*lacZ* exhibits only a slightly elevated level in the peripheral region of group 1, but becomes higher in the peripheral compared to the central region of group 3 (see S7 Fig for raw images). (G) Ratios of Dll/Lim1 and Delta/Serrate from single cells are plotted for group 1 (green, N = 49), group 2 (orange, N = 50), and group 3 (black, N = 51). Regression lines from the three stages show a positive correlation, with increasing correlations over development time (compare *R*^*2*^ indicated next to the regression lines). Scale bars: 50μm.

Since Notch (N) signaling is involved in the wing D/V boundary, mediated through intercellular actomyosin cables, we checked whether N might also be involved in formation of the A1 fold. N activity, based on anti-N^intra^ and N reporters *E(spl)mβ-lacZ* and *Su(H)Gbe-lacZ*, showed ring like patterns in l-L2 antenna discs (Fig 6A, 6C and 6E). In e-L3, N activity was enhanced in the A1 fold (Fig 6B and 6D). We examined in detail the relative timing of segregated expression of Dll, Lim1 and the N ligands, Delta (Dl) and Serrate (Ser) in l-L2. Disc sizes in groups 1, 2, and 3 (see material and methods) were 4835 ± 328, 6058 ± 231, and 7065 ± 309μm^2^ (mean ± stdev), respectively. Dl was mostly expressed in the central region, whereas Ser was expressed at the periphery of discs (S7A–S7C Fig cross-sections). These patterns echo the expressions of Dll and Lim1 during l-L2, respectively (Fig 6F). Lim1/Dll segregation is apparent in group 1, whereas segregated expression of Dl/Ser begins later in group 2 and is more pronounced in group 3 (Fig 6F; S7A’–S7C’ Fig). Fringe (Fng) is a glycosyltransferase that can modulate the interaction between N and its ligands [54]. Timing of *fng*-*lacZ* differential expression correlated with Dl/Ser segregation (Fig 6F and S7D’–S7F’ Fig). We further analyzed expression levels of these proteins in single cells from different groups. The correlation of Dll/Lim1 segregation and Dl/Ser segregation within single cells increased significantly with increasing disc size (Fig 6G). Taken together, these results suggest that sharp segregation of Dll and Lim1 expression precedes the differential expression of Ser, Dl and Fng, and thereby define the localization of N activation.

**Fig 7:**
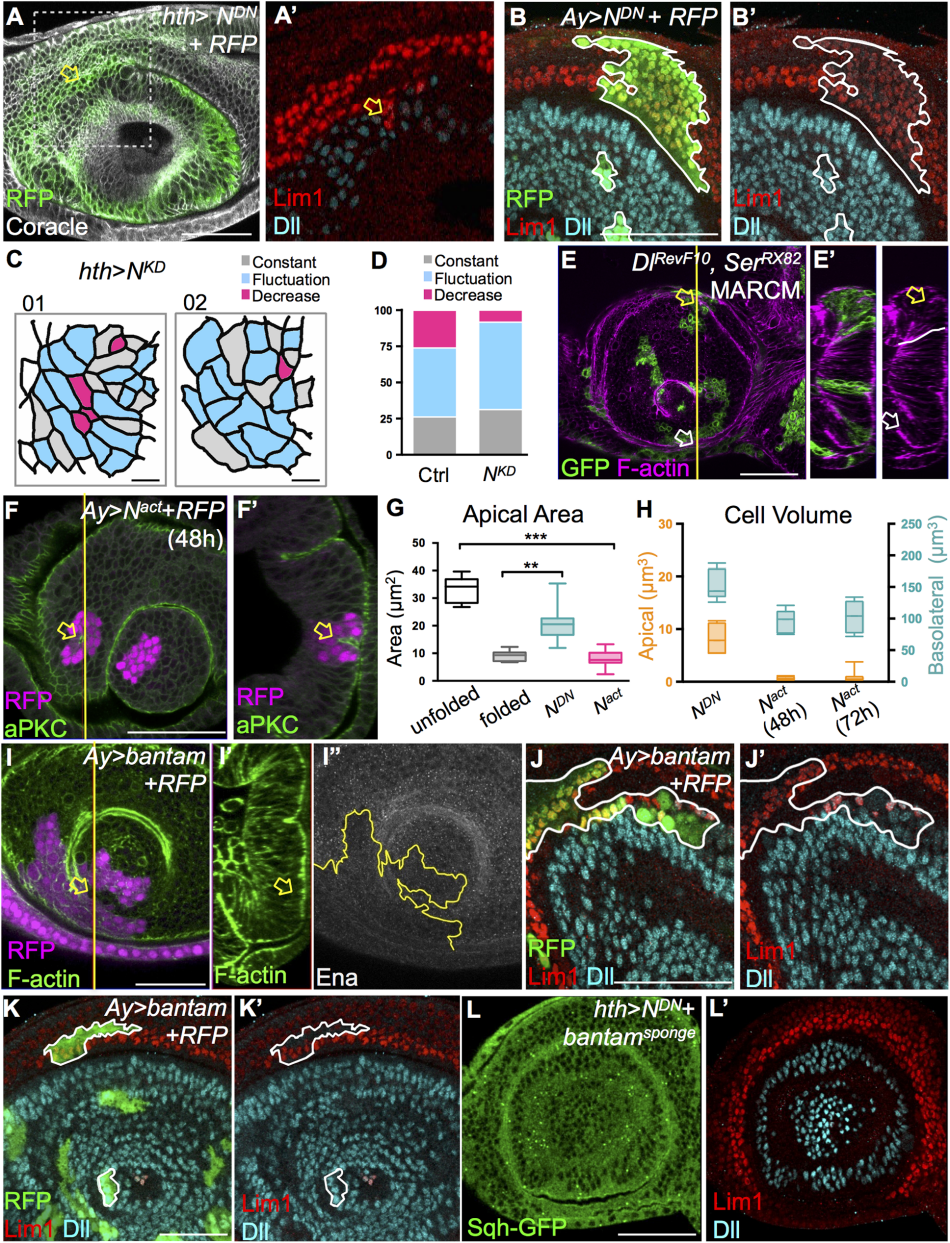
Notch activation drives apical constriction and epithelial fold. (A) Dominant-negative Notch (*N*^*DN*^) expression by *hth*-*GAL4* disrupts the A1 fold (arrow, Coracle, white; RFP, green) and mixing of Lim1 (red) and Dll (blue) cells (A’: enlargement of boxed region in A, 25/29). (B) *N*^*DN*^ clones within Dll or Lim1 single fields do not alter cell fate (14/14, compare Dll and Lim1 intensity in *N*^*DN*^ and in control cells). (C) *Ex vivo* observation of N RNAi driven by *hth*-*GAL*4 (*hth*>*N*^*KD*^) in the EAD. Changes in cell apical size were monitored by Sqh-GFP. The three types of cells (decreasing, fluctuating and constant, see Fig 3B and 3C) are color-coded. Still frames from two samples at T_final_ are shown. The A1 fold fails to form in the absence of N signaling. (D) Proportions of the three cell populations in *hth*>*N*^*DN*^ EAD. There is a reduction of cells with a decreasing apical area (red), and an increase of cells with a fluctuating apical area (blue). Cell numbers of constant, fluctuating, and decreasing groups are 17, 31, and 17 in the control, and 15, 29, and 5 in *N*^*KD*^. ((E-E’) *Dl*^*RevF10*^, *Ser*^*RX82*^ MARCM clones (mutant cells marked by mCD8-GFP, green) show reduced A1 fold (E’: compare white and yellow arrows in the control and mutant cells, respectively, in the optical section, 14/18). White line indicates a clone border. (F-F’) Clonal expression of *N*^*act*^ (RFP, magenta) causes ectopic fold (arrow) within the clone, even when away from the fold (37/49). (F’) Optical section along the yellow line indicated in (F). (G) The effect of N activity on cellular apical surface area. Cells with constitutively-active (*N*^*act*^, N = 23) or dominant-negative (*N*^*DN*^, N = 28) N activity have apical areas similar to cells at the A1 fold (fold cells, N = 11) or cells outside of the fold (non-fold cells, N = 21), respectively. (H) The volumes of the apical and basolateral domains (defined by aPKC and FasIII, respectively) in cells expressing *N*^*act*^ or *N*^*DN*^ (N = 12) were quantified. *N*^*act*^ cells were assayed at 48h (N = 16) and 72h (N = 14) post-clonal inductions. (I-K) Clonal expression of *bantam* (marked by RFP, red in I; green in J-K). The clone border is marked by a yellow line (I”) or a white line (J-K). (I) *bantam* blocks epithelial fold (arrow) via reduced Ena (I’, white) within the clone (16/22). (J) *bantam* overexpression results in mixing of Lim1 and Dll cells within the clone (9/14). (L) Coexpression of *N*^*DN*^ and *bantam*^*sponge*^ by *hth*-*GAL4* shows normal A1 fold and no mixing of Lim1/Dll (L’, 10/13). Scale bars: 50μm, except in C: 5μm. ** P ≤ 0.01 *** P ≤ 0.001 (ANOVA-Tukey’s multiple comparisons).

N is activated during e-L3 at the A1 fold, hence we tested whether N signaling is responsible for the epithelial fold formation. An N dominant-negative (*N*^*DN*^) mutant was expressed by *hth*-*GAL*4 and examined at l-L3. The A1 fold of this mutant was disrupted with high penetrance (Fig 7A, arrow, 86%) and was always accompanied by mixing of Dll and Lim1 cells (Fig 7A’). *N*^*DN*^ clones that did not span the A1 fold presented normal Dll and Lim1 expressions (Fig 7B–7B’), indicating that reduced N activation did not alter cell fates. The cell morphology of *ex vivo*-cultured *hth>N*^*KD*^ EAD was monitored by Sqh-GFP for more than 5 hours (Fig 7C–7D, compare to Fig 3B). These EAD failed to form the A1 fold. Most antennal cells exhibited a fluctuating apical area (Fig 7C, blue cells; 7D, quantitation), and a few scattered cells underwent apical constriction (Fig 7C, red cells). Since the N ligands Dl and Ser are differentially expressed in the Dll and Lim1 domains, respectively, we generated *Dl*^*RevF10*^ *Ser*^*RX82*^ double mutant clones so that for any clone spanning the A1 fold, no N ligand could activate Notch. Indeed, in such mutant clones, the A1 fold failed to form (Fig 7E, compare white and yellow arrows). Cells in the *N*^*DN*^ clones showed less apical constriction (Fig 7G), and the cell volumes of their apical and basolateral domains were similar to those of non-folded cells (Fig 7H, compare with Fig 3E). These results indicate that N signaling is required for the formation of the A1 fold. In contrast, clonal expression at l-L2 of constitutively-activated N (*N*^*act*^, the Notch intracellular domain [55]) caused ectopic tissue fold when located in a non-fold region (Fig 7F–7F’, arrow, 76%). The cells at the ectopic fold showed apical constriction and reduced apical and basolateral volumes similar to cells at the A1 fold (Fig 7G, 7H, and Fig 3E). The reduced cell volume in *N*^*act*^ cells is likely due to shrinkage, since the volume of these cells in L3 (Fig 7H) is smaller than normal cells in L2 (Fig 3E). Prolonged N activation did not cause further changes in cell volume (Fig 7H, compare 48h and 72h), suggesting that these drastic changes in cell morphology were stable upon N activation. Together, these loss-of-function and gain-of-function results show that N signaling drives the formation of stable tissue folds in the antennal disc.

N signaling is important for the establishment of the D/V boundary in wing disc [56, 57]. There, it represses the micro-RNA *bantam*, which itself represses its target Enabled (Ena), that is a positive regulator of actin polymerization. By repressing *bantam*, N enhances Ena expression, thereby establishing the actomyosin cable-based D/V boundary [19]. We assessed endogenous *bantam* level by RNA *in situ* hybridization in combination with a N activity reporter, *Su(H)Gbe*-*lacZ*, and Ena to study their relative expressions in the EAD. The *bantam* RNA *in situ* signals recapitulated the patterns reported previously in the wing D/V boundary [19] (S8A Fig). In l-L2 antennal discs, *bantam* and Ena levels were generally low, with little correlations with *Su(H)Gbe*-*lacZ* level (S8C Fig). In e-L3, a relatively lower *bantam* level was observed in the A1 fold region, whereas *Su(H)Gbe*-*lacZ* and Ena level were both elevated (S8D and S8D” Fig arrows). *bantam*-overexpressing clones showed significantly reduced Ena levels and inhibited EAD fold (Fig 7I), as well as mixing of Lim1 and Dll cells (Fig 7J). The *bantam*-overexpression clones within a single field did not exhibit altered Lim1 and Dll expression, indicating that the cell mixing phenotype was not the result of a changed cell fate (Fig 7K). Concomitant blocking of N signaling (through *N*^*DN*^) and a reduction of *bantam* (by expressing *bantam*^*sponge*^) in *hth>N*^*DN*^*+bantam*^*sponge*^ mutants rescued the disrupted A1 fold and the lineage mixing phenotype (Fig 7I and 7K, 23% phenotype, compared to 81% in *hth>N*^*DN*^ in Fig 7A). The Notch/*bantam* axis has been shown to regulate cell proliferation and apoptosis [58, 59]. We further tested if such regulation also exists and may potentially affect A1 fold formation. Mitosis (phospho-Histone H3) and apoptosis (cleaved caspase 3, S8E and S8F Fig) were examined in *N*^*DN*^ or *bantam* overexpression mutants driven by *dpp-GAL4* from L2 (*dpp*^*L2*^). Cell proliferation was reduced by about 30% in both mutants, whereas there were no significant changes in apoptosis. In contrast to the nearly complete absence of proliferation reported in the DV boundary of wing disc [17, 60], this 30% reduction may not significantly affect the formation of epithelial folds. Our results suggest that N acted through *bantam* and Ena (possibly by repressing *bantam* to allow Ena expression) to induce actomyosin assembly and thus epithelial constriction and formation of the A1 fold.

### Folded epithelial structures reinforce N signaling

**Fig 8:**
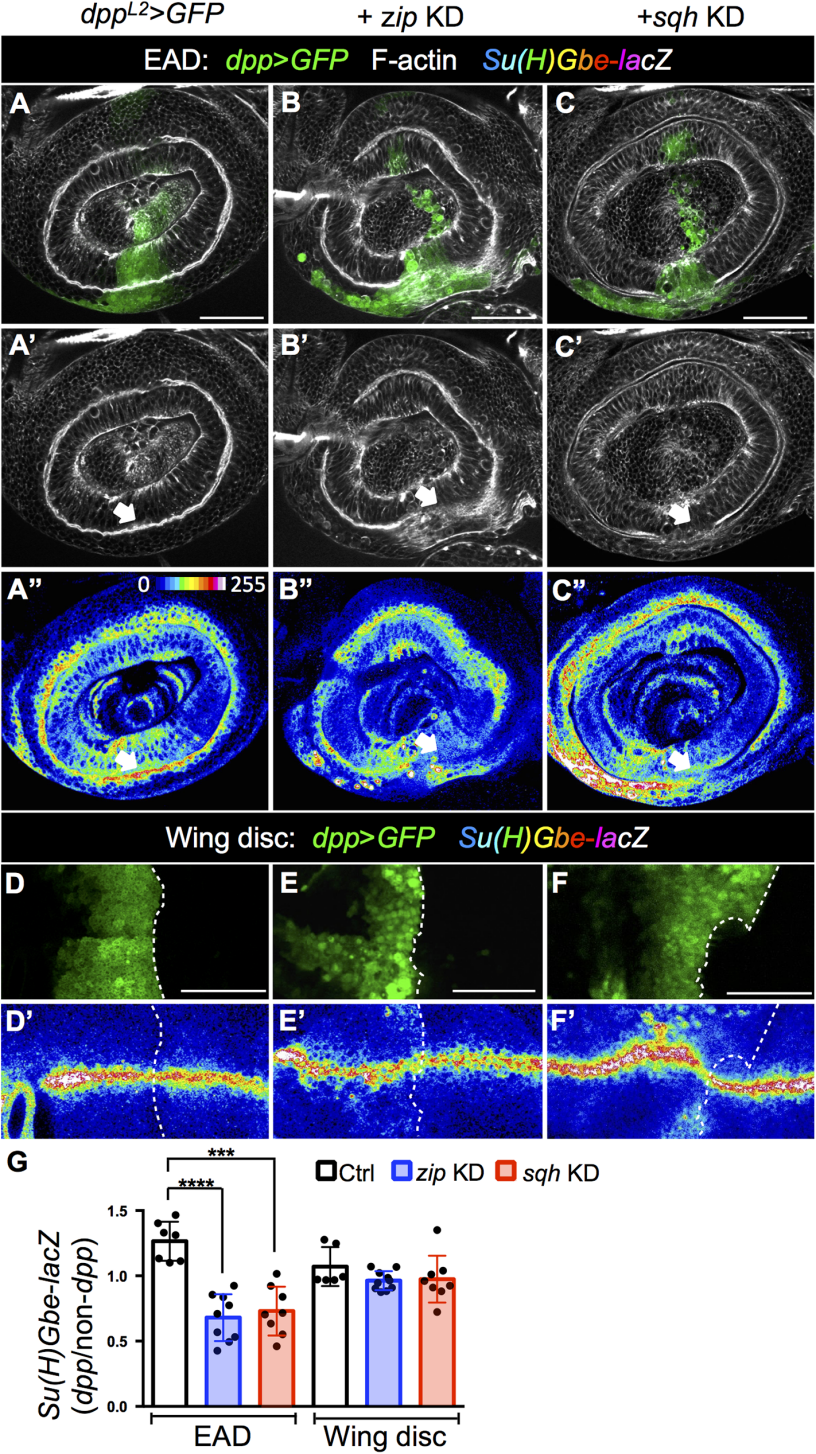
The folded epithelial structure reinforces N signaling. N activity is indicated by the reporter *Su(H)Gbe-lacZ* (in heat map). The epithelial fold is marked by F-actin (white). (A-C) EAD. (D-F) Wing disc. (A, D) The *dpp* expression domain in the EAD (A) and wing disc (D) is indicated by GFP expression (*dpp*^*L2*^>GFP, green). *zip* (B,E) and *sqh* (C, F) were individually knocked down from L2. Knockdown of actomyosin specifically disrupted A1 fold in the *dpp* region (B’ and C’, arrow) and reduced *Su(H)Gbe-lacZ* levels in the EAD (B”, C”) but not in wing disc (E’, F’). (G) The level of *Su(H)Gbe-lacZ* in *dpp-GAL4* was normalized with a non-*dpp* region in the respective EAD or wing disc. Total analyzed disc numbers in control, *sqh* KD, and *zip* KD were 7, 8, and 9 (for EAD) and 6, 8, and 10 (for wing disc) respectively. Scale bars: 50μm. *** P ≤ 0.001 **** P ≤ 0.0001(ANOVA-Dunnett’s multiple comparisons).

Even after formation of the A1 fold, N activity is sustained during e-L3 (Fig 6D). We found that blocking epithelial fold, by knock down of *zip* and *sqh* in the *dpp* expression domain that spans the dorsal A1 fold reduced the expression level of the N reporter *Su(H)Gbe*-*lacZ* (Fig 8A–8C; 8G, quantitation). Interestingly, levels of the same N reporter were not affected in conditions where D/V boundaries were disrupted in wing discs (Fig 8D–8F; 8G). This suggests that N activity is sustained by the epithelial fold, possibly representing positive feedback regulation.

## Discussion

### The temporal and causal sequences in boundary formation

**Fig 9:**
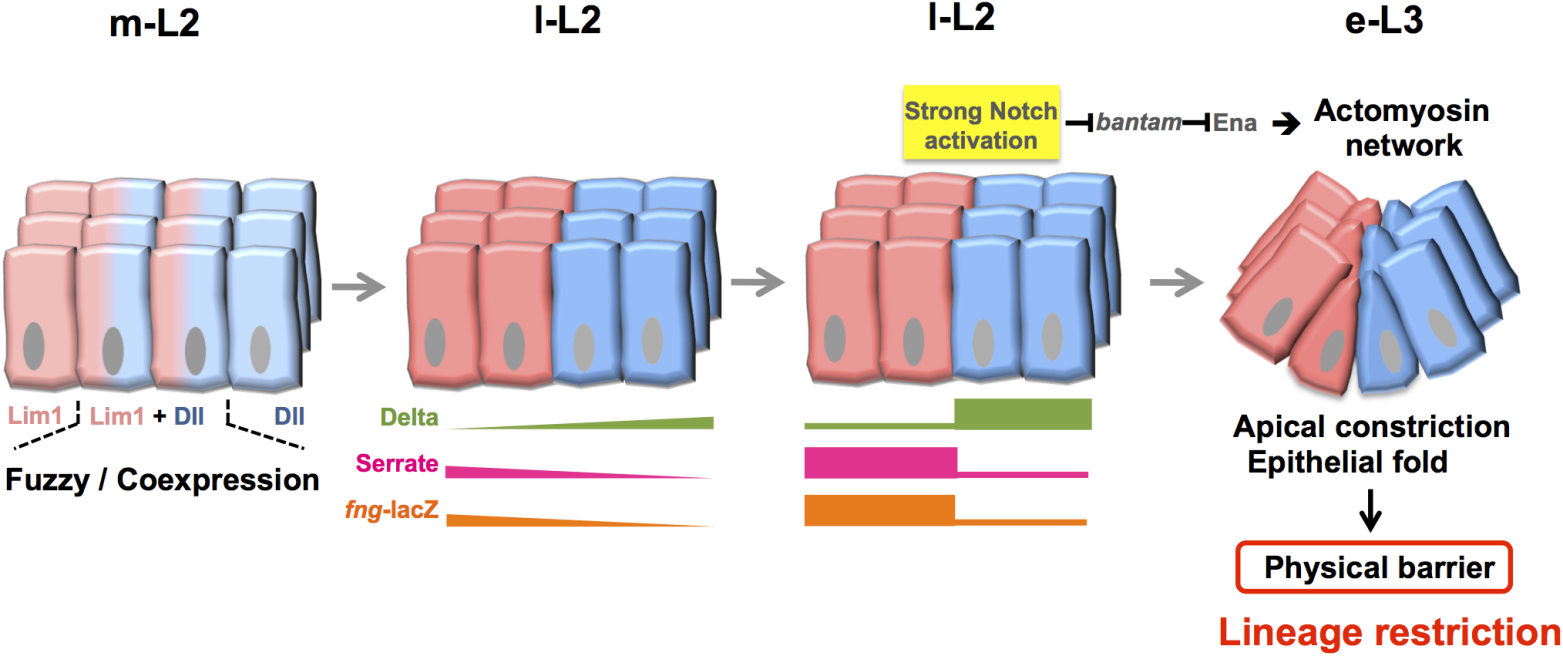
The process of A1 boundary formation. At m-L2, expression of the Dll and Lim1 selector genes in cells is initially low, with fuzzy coexpression. From l-L2 to e-L3, expression of both Dll and Lim1 becomes elevated and sharply segregated. This is followed by differential expression of Dl in the Dll domain, and Ser and Fng in the Lim1 domain, and then subsequently by N activation at the Dll/Lim1 interface. N activity then represses *bantam*, resulting in the de-repression of Ena, which triggers non-cable actomyosin-dependent cytoskeleton reorganization to drive apical constriction and epithelial fold. The epithelial fold then serves as a physical barrier to prevent mixing of cells from the Dll and Lim1 field.

In this study, we tried to unravel the molecular and cellular mechanisms of boundary formation in the *Drosophila* head. We focused our analysis on the antennal A1 fold that separates the A1 and A2-Ar segments. Our results showed that the expression of the selector genes *Lim1* and *Dll*, which are expressed in A1 and A2-Ar, respectively, was sharply segregated. This step was followed by differential expression of Dl, Ser and Fng, as well as activation of N signaling at the interface between A1 and A2 (Fig 9). N signaling then induced apical constriction and epithelial fold, possibly through repression of *bantam* to allow levels of the *bantam* target Ena to become elevated, with this latter inducing the actomyosin network. The actomyosin-dependent epithelial fold then provided a mechanical force to prevent cell mixing. When N signaling or actomyosin was disrupted, or when *bantam* was overexpressed, the epithelial fold was disrupted and Dll and Lim1 cells become mixed. Thus we describe a clear temporal and causal sequence of events leading from selector gene expression to the establishment of a lineage-restricting boundary.

Sharp segregation of Dll/Lim1 expressions began before formation of the A1 fold, suggesting that fold formation is not the driving force for segregation of Dll/Lim1 expression. Instead, the fold functions to safeguard the segregated lineages from mixing. Whether Dll/Lim1 segregated expression is due to direct or indirect antagonism between the two proteins is not known.

### Actomyosin-dependent apical constriction and epithelial fold is a novel mechanism for boundary formation

Actomyosin-dependent apical constriction is an important mechanism for tissue morphogenesis in diverse developmental processes, *e*.*g*. gastrulation in vertebrates, neural closure and *Drosophila* gastrulation, as well as dorsal closure and formation of the ventral furrow and segmental groove in embryos (see reviews [61, 62]). Our study describes a new function of actomyosin, *i*.*e*., the formation of lineage-restricting boundaries via apical constriction during development.

This actomyosin-dependent epithelial fold provides a mechanism distinctly different from other known types of boundary formation. We found that the cells at the A1 fold still undergo mitosis, suggesting that mitotic quiescence is not involved. Perhaps epithelial fold as a lineage barrier is needed in situations in which mitotic quiescence does not happen. Mechanically and physically, epithelial folds could serve as stronger barriers than intercellular cables when mitotic activity is not suppressed. The drastic and sustained morphological changes, including reduced apical area and cell volume, may be accompanied by increased cortical tension of cells along the A1 fold [63, 64], with such high interfacial tension then preventing cell intermingling and ensuring Dll and Lim1 cell segregation [30, 65]. Although similar to actomyosin boundaries, the epithelial fold in the A1 boundary is distinctly different from the supracellular actomyosin cable structure in fly parasegmental borders, the wing D/V border, and the interrhombomeric boundaries of vertebrates [19, 25–27] (see review [66]). The adherens junction protein Ed, which is known to promote the formation of supracellular actomyosin cables [50], is not involved in A1 fold formation (S5G Fig). Although actomyosin is enriched in a ring of cells in the A1 fold, it does not exert a centripetal force to close the ring, unlike the circumferential cable described in dorsal closure and wound healing (see review [67]). In the A1 fold, the constricting cells become smaller in both their apical and basolateral domains, thus differing from ventral furrow cells where cell volume remains constant [68, 69].

A tissue fold probably provides a strong physical or mechanical barrier to prevent cell mixing. In addition, whereas in a flat tissue where the boundary involves only one to two rows of cells, the tissue fold involves more cells engaging in cell-cell communication. The close apposition of cells within the fold may allow efficient signaling within a small volume [70]. This may be an evolutionarily conserved mechanism for boundary formation that corresponds to stable morphological constrictions such as the joints in the antennae and leg segments (see below).

### Notch signaling participates of stable boundary formation

Although N signaling has been reported to be involved in many developmental processes, a role in inducing actomyosin-dependent apical constriction and epithelial fold is a novel described function for N. For the A1 boundary, N activity is possibly mediated through repression of *bantam* and consequent upregulation of Ena. In the wing D/V boundary, N signaling is also mediated through *bantam* and Ena, but the outcome is formation of actomyosin cables, *i*.*e*., without apical constriction and epithelial fold [19]. Thus, the N/*bantam*/Ena pathway for tissue morphological changes is apparently context-dependent.

Tissue constriction also occurs later in joint formation of the legs and antennae. N activation also occurs in the joints of the leg disc and is required for joint formation [71–74]. This role is conserved from holometabolous insects like the fruitfly *Drosophila melanogaster* and the red flour beetle *Tribolium castaneum* [75] to the hemimetabolous cricket *Gryllus bimaculatus* [76]. It is possible that for segmented structures that telescope out in the P/D axis, like the antennae, legs, proboscis and genitalia, N signaling is used to demarcate the boundaries between segments, which are characterized by tissue constriction. N-dependent epithelial fold morphogenesis has also been reported in mice cilia body development without affecting cell fate [77], suggesting that such N-dependent regulation in morphogenesis is evolutionarily-conserved.

We propose that N signaling is important in all boundaries that involve stable tissue morphogenesis. For those boundaries corresponding to stable morphological constrictions, *e*.*g*. the joints in insect appendages, N acts via actomyosin-mediated epithelial fold. The wing D/V boundary represents a different type of stable tissue morphogenesis. It becomes bent into the wing margin and involves N signaling via actomyosin cables, rather than apical constriction. In contrast, actomyosin-dependent apical constrictions do not involved N signaling and are involved in transient tissue morphogenesis, such as gastrulation in vertebrates, neural closure, *Drosophila* gastrulation, dorsal closure, as well as formation of the ventral furrow, eye disc morphogenetic furrow, and segmental groove in embryos (see review [61]).

N signaling is also involved in the boundary between new bud and the parent body of *Hydra*, where it is required for sharpening of the gene expression boundary and tissue constriction at the base of the bud [78]. Whether the role of N in these tissue constrictions is due to actomyosin-dependent apical constriction and epithelial fold is not known.

### Boundary stability and maintenance

Boundaries may be established early in development. As the tissue grows in size through cell divisions and growth, boundary maintenance become essential. We found that N activity is maintained by actomyosin, suggesting feedback regulation to stably maintain the boundary. Mechanical tension generated by actomyosin networks has been suggested to enhance actomyosin assembly in a feedback manner (see review [79]). Interestingly, the N-mediated wing A/P and D/V boundaries, which form actomyosin cables rather than tissue folds, did not exhibit such positive feedback regulation (Fig 8D–8F). Instead, the stability of the *Drosophila* wing D/V boundary is maintained by a complex gene regulatory network involving N, Wg, N ligands and Cut [80, 81]. Perhaps this is necessary for a boundary not involving tissue morphogenesis.

### The A1 fold is a boundary between the coxopodite and telopodite

The segmented appendages of arthropods (antennae, legs, mouth parts) are homologous structures of common evolutionary origin ([82, 83]). Snodgrass (1935) proposed that the generalized arthropod appendage is composed of a proximal segment called the coxopodite and a distal segment called the telopodite, either of which can further develop into more segments. The coxopodite is believed to be an extension of the body wall, whereas the telopodite represents the true limb, and thus represents an evolutionary addition [84, 85]. *Dll* mutants lack all distal segments except for the coxa in legs and the A1 segment in antennae [84, 86, 87]. Lineage tracing studies have shown that Dll-expressing cells contributed to all parts of the legs except the coxa [87, 88]. These results indicate that the leg coxa and antenna A1 segment correspond to the Dll-independent coxopodite, and that Dll is the selector gene for the telopodite. Therefore, the antennal A1 fold is the boundary between the coxopodite and telopodite. We postulate that the same N-mediated epithelial fold mechanism also operates in the coxopodite/telopodite boundary of legs and other appendages.

## Materials and methods

### Fly stocks

Flies were cultured in 25°C according to standard procedure unless otherwise noted. *w*^*1118*^ larvae were used for expression pattern analysis. Fly stocks were: *sqh*^*AX3*^*; sqh*-*SqhGFP42* (Sqh–GFP) [89], Moe-ABD::GFP (also known as sGMCA [53]) was from Dan Kiehart (Duke University, North Carolina), *hth*-*GAL4* [90] was from Richard Mann (Columbia University, New York), *tub*-*GAL4* [91] was from Tzumin Lee (Janelia Farm Research Campus, HHMI, Virginia), *dpp*-*GAL4*^*c40*.*6*.^ was from Jessica Treisman (New York University), *fng*-*lacZ* [92], *Su(H)Gbe*-*lacZ* was from Sarah Bray (University of Cambridge, UK), *E(spl)mβ*-*lacZ* [93], *UAS*-*N*^*act*^ [55], *UAS*-*N*^*DN*^ [94], *UAS*-*bantam* and *UAS-bantam*^*sponge*^ [19] were from Marco Milán (Institute for Research in Biomedicine, Barcelona). *UAS*-*RNAi* stocks were from VDRC (*zip*: 7819, *sqh*: 7916, *mys*: 29613, *rhea*: 40399, *jub*: 38442, *ed*: 104279/3087), NIG (*N*: 3936-R2), and Bloomington (*N*: 7870, *zip*: 36727, *sqh*: 32439, *mys*: 27735, *rhea*: 28950).

Genotypes for the mutant and MARCM clonal analysis were: *hs*-*FLP*^*1*^; *UAS*-*rCD2*-*RFP*, *UAS*-*miR*-*GFP*, *FRT*^*40A*^/ *UAS*-*mCD8*-*GFP*, *UAS*-*miR-CD2*, *FRT*^*40A*^; *tub-GAL4*/+ [41], *hs-FLP*; *FRT*^*42B*^, *zip*^*2*^/*FRT*^*42B*^, *ubi-GFP*, *hs-FLP*; *FRT*^*42B*^, *zip*^*2*^/*FRT*^*42B*^, *tub-GAL80; tub-GAL4/ UAS-GFP [48]*, *hs-FLP*; *FRT*^*42D*^, *sqa*^*f01512*^/*FRT*^*42D*^, *ubi-GFP* (DGRC114526, *sqa*^*f01512*^ is a *PiggyBac* insertion in *sqa* [95]), *hs-FLP*; *tub-GAL4*, *UAS-mCD8GFP*/+; *FRT*^*82B*^, *Dl*^*RevF10*^, *Ser*^*RX82*^/*FRT*^*82B*^, *tub-GAL80* (Bloomington 6300).

### Clone induction and animal staging

Positive labeled clones were induced using *hs-FLP*^*122*^*; +; Act5C>CD2>GAL4*, *UAS-RFP* [96]. Induction of *hs-FLP*^*122*^ was conducted at 38°C for 8 min at 24 or 48h after egg-laying (AEL). For lineage tracing experiments using Twin-Spot MARCM [41], newly-hatched first instar larvae were collected every two hours from juice plates, and kept in 25°C before heat shock (38^°^C for 10min). Larvae were raised under conditions of 25^°^C except for heat-shock at the indicated stage. Clonal induction was performed at L1 (AEH 18-20h), mL2 (AEH 26-28h), l-L2 (AEH 38-40h), or e-L3 (AEH 48-50h) stage. The discs were dissected and examined at l-L3.

We use AEH (after egg-hatching) for Twin-spot MARCM, and Dll/Lim1 expression pattern analysis, for which more precise timings are required. AEL (after egg-laying) was used for genomic mutant (*zip*^2^, *sqa*^*f01512*^, and *Dl*^*RevF10*^, *Ser*^*RX82*^, induced at L1), *Ay* (induced at L1/ L2), and *tub*-*GAL80*^*ts*^ experiments.

### Immunohistochemistry

Antibody staining was performed according to a procedure described previously [36]. Primary antibodies from DSHB (Developmental Studies Hybridoma Bank, University of Iowa) were mouse-anti-Coracle (C615.16, 1:20), mouse-anti Cut (2B10, 1:100), mouse-anti-Dl (C594.9B, 1:300), mouse-anti-Dlg (4F3, 1:200), mouse-anti-Ena (5G2, 1: 100), mouse-anti-FasIII (7G10, 1:50), mouse-anti-GFP (12A6, 1:100), mouse-anti-N^intra^ (C17.9C6, 1:200), mouse-anti-Ptc (Apa1, 1:100). Other primary antibodies included rabbit anti-Lim1 (1:400, from Dr. Juan Botas), rat-anti-Serrate (1:1000, preabsorbed, from Dr. Kennith Irvine), rabbit anti-aPKC (C-20, 1:50, Santa Cruz), rabbit-anti-caspase3 (cleaved) (1:200, Cell Signaling), goat-anti-Dll (F-20) (1:100, Santa Cruz), rabbit-anti-GFP (1:1000, Invitrogen), rabbit-anti-β-gal (1:5000, Cappel), rabbit-anti-phospho-Histone 3 (1:200, Millipore), rat-anti-RFP (5F8) (1:1000, Chromotek), Phalloidin (F-actin, Alexa 488-/555- or 647-conjugated) (1:100, Life Technologies). Species-matched Alexa 488-/561- or 633-conjugated secondary antibodies were from Jackson ImmunoResearch. Alexa Fluor 405-donkey anti-rabbit was from Abcam (ab175651). Images were acquired using a Zeiss LSM 780 or 710 with appropriate GaAsP detectors. Objectives were Plan-Apochromat 20x/0.8, Plan-Apochromat 40x/1.4 Oil, C-Apochromat 40x/1.2W Korr, and Plan-Apochromat 63x/1.4 Oil (Zeiss). All the images in this study were oriented dorsal-face up and with the posterior end to the right. Optical sections were oriented with the apical face of the disc proper to the right or top.

### Image processing and quantitative analysis

Images were processed with ZEN (Zeiss) with minimal brightness/contrast adjustments. To analyze the pixel intensities of Dll, Lim1 and N related (Dl, Ser and *fng-lacZ*) expression patterns, optical sections of 60μm were manually positioned with the center (0 in the X axis) placed at the fold (eL3) or at the Dll-Lim1 overlapping regions (L2). Although the larvae were collected at 1 hour intervals, there were still variations in developmental timing. Therefore, more than ten EADs were imaged, and only those of similar size were chosen for further analysis. Disc sizes in groups 1, 2, and 3 were, respectively: 4835 ± 328, 6058 ± 231, and 7065 ± 309μm^2^ (mean ± stdev). More than five EADs were quantified and 2–3 optical sections were analyzed per EAD. The signal intensity was established from the histogram analysis module in ZEN (Zeiss) and normalized to the basal level in non-expressing cells. The center (0 in the X axis) was manually positioned at the center of the Dll-Lim1 overlapping region. Correlations of ratios between Dll/Lim1 and Delta/Serrate were achieved by individual mean intensities from single cells. The stack images of 16–18μm were projected to ensure coverage of ligands and to identity genes. Cells with Dll-only, Dll+Lim1, and Lim1-only expressions were collected from the three groups. To establish *Su*(*H*)*Gbe-lacZ* levels in *sqh* and *zip* knockdown experiments, the pixel intensity of *lacZ* from optical sections across the A1 fold was quantified using the average pixel intensity of *dpp*-expressing regions normalized with non *dpp*-expressing regions in the same discs.

Time-lapse imaging to track cell morphology (Sqh-GFP and Sqh-mCherry) from l-L2 EAD *ex vivo* cultures was processed in Imaris software (Bitplane). 3D-projected images from time-lapse stacks were acquired using the surpass mode. A total of 5 hours of stack images were rotated and cropped in 3D to remove the peripodial membrane and basolateral regions. Segmentation of individual cells was carried out using the filament module with minimal manual corrections. The surface module was further applied to the post-filament images to obtain cell sizes and automatic tracking over time. Each cell was pre-processed for its absolute apical area value over time to determine whether it belonged to the constant (δArea < 10μm^2^), fluctuating (δArea ≥ 10μm^2^), or decreasing (initial apical area around 20–40μm^2^, and final < 10μm^2^) groups. For individual cells (as indicated by “i”), apical areas at each time point (A_ti_) were subtracted from the respective mean area over time (A_avgi_) before normalization with the respective mean ((A_ti_-A_avgi_)/A_avgi_) to represent the proportional change. Proportional changes of cells in the same group were plotted as total mean and stdev. Trajectories of RFP clones in Sqh-GFP were accessed by spot tracking module in Imaris software (S2 and S3 Movie). The spot detection diameter was set to 1μm (shown as center point), with maximum distance between time points for 2μm. Autoregression motion algorithm were used to track RFP signal over time. 3D surpass time-lapse images were shown in spot center point with trajectory in dragon tail mode (for 20 time points). The overall trajectories of individual cells were presented in color-coded time map.

Apical (aPKC) and basolateral (FasIII) cell volumes were acquired from serial sections of fixed EAD using the Imaris surface module. Individual cell contours along the XY plane were outlined using the autofit module through all stack images, with settings of full accuracy and least impact. Stack contours from single cells were further processed to generate a 3D surface render and to acquire apical and basolateral volumes. For the basolateral domain, pinhole = 0.9 μm, optical interval = 0.47μm (total z = 30–40μm). For the apical domain, pinhole = 0.5 μm; optical interval = 0.27 μm (total z = 4–7μm).

Data sets were analyzed and plotted in Prism 6 using two-tailed un-paired t tests (S2C Fig, S6C Fig), linear regression analyse (Fig 6G), ANOVA-Tukey’s multiple comparisons (Fig 7G), and ANOVA-Dunnett’s multiple comparisons (Fig 8G; S5M Fig; S8E and S8F Fig).

### Scanning electron microscopy

Adult flies were fixed in Bouin’s solution, followed by serial dehydrations in 25%, 50%, 75%, and then 100% ethanol solutions before being transferred to 100% acetone. The samples were further processed by critical-point drying with liquid CO_2_, followed by sputter-coating with gold. Images were acquired using an Environmental Scanning Electron Microscope (FEI Quanta 200).

### *Ex vivo* culture of EAD

*Ex vivo* culturing and live imaging of EAD were as described [46]. For l-L2 and e-L3 EAD, the discs were embedded in 0.6% and 0.75% low gelling agarose, respectively.

### Chromophore assisted laser inactivation (CALI) treatment

Sqh-GFP and Moe-ABD::GFP were used as target molecule for CALI. CALI was carried out using an LSM710 inverted confocal microscope (Zeiss) with a 488nm laser (25mW) set at 100% of its power for a total of five cycles with 300 iterations per cycle (20–25 minutes break between each cycle, total of CALI treatment for 2.5 hours). The numerical zoom was set to 5 using a 40x objective. The region for CALI treatment was 3μm x 20μm with differential Z adjusted manually each time. Time-lapse images were acquired pre- and post-CALI treatment, with Z-stack set to a mean of 35μm. The time interval between each stack was 6 min as indicated in the S2 Movie. The parameters were: scan speed: 6 arbitrary units; number of scans per frame: 1; scanning: bi-directional; pinhole: 1.2μm; objectives: C-Apochromat 40x/1.2W Korr (Zeiss).

### FISH and protein co-staining

The EAD was dissected in DEPC-PBS, followed by fixation (4% PFA and 1% DMSO in PBS) for 20 min. Samples were washed in PBT (0.1% Tween20 in PBS) before proteinase K permeabilization (2 μg/mL in digestion buffer for 3 min, digestion buffer: 50 mM Tris-HCl, pH7.5 and 50 mM EDTA). After proteinase K inactivation (0.2% of glycine in PBS), samples were post-fixed with 4% PFA for 20 min. Samples were prehybridized in hybridization buffer (HYB: 50% formamide, 5x SSC, 0.1% Tween20, 100 μg/mL denatured salmon DNA, 100 μg/mL yeast tRNA, and 50 μg/mL heparin) for more than 1 hour at 60^°^C. The DIG-labeled probe (stock: 50 ng/μL, dilute stock 1:250 in HYB) was hybridized overnight at 60^°^C. After hybridization, samples were washed in 100% HYB, 66% HYB-PBT, 33% HYB-PBT, then PBT at 60^°^C for 1 hour each, then at room temperature for 4 more washes in PBT (5 min each). Samples were treated with 3% H_2_O_2_ in PBS to reduce endogenous HRP activity. Samples were then blocked in blocking solution (2% blocking reagent, 20% normal horse serum in PBT) for 30 min before overnight incubation with anti-Dig-HRP (POD Roche 1207–733, 1:100 dilute in blocking solution) at 4 ^o^C. TSA amplification (PerkinElmer, NEL745001KT) was used to enhance the hybridization signals before protein detection. Protein immunofluorescence was performed after RNA *in situ* hybridization as described previously [36], except all steps were conducted in the dark.

The 5’- or 3’-DIG-labeled probes for *bantam* detection and the control sequence were: aatcagctttcaaaatgatctcacttgtatg (*bantam*), and gtgtaacacgtctatacgccca (scramble-miR, EXIQON).

## Supporting information

S1 Fig Formation of epithelial folds in the EAD. (A) The progressive formation of epithelial folds (green) in the EAD at the successive developmental stages is depicted. The morphogenetic furrow (MF) in eye disc is indicated by a grey dotted line. (B-D) EAD morphological changes are revealed by F-actin (green) and Dlg (magenta, basolateral domain) staining on *w*^*1118*^. (B-B’) During l-L2, the medial epithelial cells undergo a transition from a cuboidal to columnar shape, resulting in a concave morphology in the lateral view. (B’) Z-projection of optical sections at the yellow line in (B). (C-C”) In e-L3 EAD, the antennal field showed one completed ring of folding (the A1 fold) whereas in the eye field, epithelial folding only occurred in the lateral region (dorsal (D) and ventral (V) optical sections, red arrows) but not in the medial region (M, white arrow). (D-D”) In l-L3 EAD, the epithelial fold (red arrow) between the eye and antenna fields has formed completely. The Ar fold has formed in the antenna disc. Scale bars: 50μm
(TIFF)

S2 Fig Quantitative expression of Dll and Lim1 in the EAD. Cross-sections of Dll, Lim1, and DAPI staining were analyzed for quantitative expression. Nuclei counters were obtained from DAPI signal. (A-B) Average pixel intensities of Dll and Lim1 were grouped and color-coded (red to purple, high to low) from l-L2 (A) and e-L3 (B) EAD. Cells coded in purple were considered as not expressing either Dll or Lim1. Arrows pointing to cells show co-expression of Dll and Lim1. (C) Percentages of cells co-expressing Lim1 and Dll were quantitated. Mean ± stdev of co-expression in l-L2 and e-L3 were 3.65 ± 2.38% (N = 18) and 0.27 ± 0.89% (N = 20), respectively. *** P ≤ 0.001(two-tailed un-paired t test).
(TIFF)

S3 Fig Twin-spot MARCM clones reveal the lineage-restricting boundary in imaginal discs. Sister clones are marked by GFP (green) and RFP (magenta), respectively. (A-B) 6% of discs (wing disc: 7/108, and EAD: 13/202) without heat-shock (Non-HS) showed non-specific, random GFP or RFP expressions. The non-specific signals are consistently weak, small (2–3 cells) and unpaired. (C-D) The D/V boundary (labeled by Cut expression, white) in the wing disc is not formed in L2 since clones induced at L2 cross the D/V boundary (C, 20/23). Instead, it is formed at early L3, since clones induced at early L3 do not cross the D/V border (D, 27/31). (E) Clones induced at L1 can cross the A/P boundary (marked by Ptc, white, and delineated by a yellow line) in the antennal disc. Clones induced at L2 can still cross the A/P boundary (marked by Ptc, 17/25), consistent with an earlier study showing that the A/P boundary in the antenna disc is not complete at 72h AEL [97]. (F) Clones induces at L1 are restricted by the A/P boundary in the wing disc (31/31). (G) TSM clones in adult fly head. Clones induced at the L2 crossed the boundary between head cuticle and compound eye (yellow line), indicating no lineage restriction between these two tissues at the L2 stage. Scale bars: 50μm.
(TIFF)

S4 Fig Phenotypic analysis of actomyosin mutation. (A-C) Control (A), *zip*^*2*^ (B), and *sqa*^*f0151*2^ (C) clones were analyzed for tissue morphology. Clones (no GFP, green) were induced in L1 and examined in l-L3. The optical section along the yellow line is shown on the right of each panel. In contrast to control, *zip*^*2*^ and *sqa*^*f01512*^ clones showed reduced or absent epithelial fold (compare white and yellow arrows in A, B, and C), but with normal apical-basal polarity (revealed by aPKC, magenta; FasIII, blue). (D) In *zip*^*2*^ clones (marked by absence of GFP, green), the mutant cells are enlarged. (E-E’) Some Dll cells are mislocalized (arrows) to the Lim1 domain, and can be seen at low frequency (4/23) outside of the *zip*^*2*^ mutant clone. (F) *zip*^*2*^ clones within a single field did not change the expression of Dll or Lim1. (G) In *zip*^*2*^ MARCM clones (mutant marked by GFP, green), a mixture of Dll and Lim1 (arrow) cells remained in the disc proper but were not sorted out for elimination. (H) Cleaved caspase 3-staining of *zip*^*2*^ clones. A few apoptotic cells (arrows) were detected. (I-J) Mixtures of Dll and Lim1 cells (arrow) following *zip* or *sqh* knockdown; mislocalized cells are maintained in the epithelial sheet (cross sections in I”-I”‘, J”-J’”). Scale bars: 50μm, except in D: 10μm.
(TIFF)

S5 Fig Phenotypic analysis of β-integrin (Mys), talin (Rhea), Jub and Ed mutation. (A-H) Cell morphology (F-actin, green) and A1 fold (arrow in the Z-axis projection along the yellow line) were examined following knockdown of specific proteins in the proximal domain that encompass the A1 boundary, driven by *hth*-*GAL4* (marked by RFP, magenta). (A-D) Knockdown of β-integrin (*mys*, A-B), or talin (*rhea*, C-D) causes cell enlargement, presumably due to a lack of basal focal adhesion, but did not affect the A1 fold. The Dll (blue) and Lim1 (red) domains remained sharply segregated (B and D). (E-H) Knockdown of *jub* (E-F) or *ed* (G-H) does not affect the A1 fold or Dll/Lim1 segregation. (I-L) Cell enlargement and/or delamination (indicated by stars) in *sqh* (I), *mys* (J), *jub* (K), and *ed* (L) knockdown mutants. (M) For each cell, serial focal planes were examined and the maximum circumference was selected for quantification. The average circumferences from single cells in different genotypes were compared. Ctrl. (mean ± stdev): 11.61 ± 2.36 (N = 21); *zip* KD: 23.37 ± 4.86 (N = 17); *sqh* KD: 22.94 ± 4.24 (N = 18); *mys* KD: 21.63 ± 3.45 (N = 21); *jub* KD: 24.9 ± 5.17 (N = 19); *ed* KD: 24.86 ± 4.61 (N = 20). Scale bars: 50μm, except in I-L: 10μm.
(TIFF)

S6 Fig Post CALI characterization on disc morphology and cell trajectory analysis. (A-B) Post-CALI (boxed region) EAD cultured for additional 6 (A) or 14 (B) hours were examined for the A1 fold (arrow in cross section) and gross morphology via Sqh-GFP (green) and Coracle (white) staining. (C) Overlay of cell trajectories in the CALI (for cells that crossed the A1 fold, N = 5) and non-CALI (N = 13) region. Displacements from T_0_ position (aligned in the center, for CALI = post CALI 0:00:00) are drawn. Each color line represents one cell. The average tracking time was 12 hours. The orientation and displacement in the *x* and *y* axis were not significantly different between the cells in the CALI and non-CALI (two-tailed un-paired t test). Scale bars: 50μm.
(TIFF)

S7 Fig Dll, Lim1, Delta, Serrate, and *fng*-*lacZ* expressions are highly correlated before tissue fold at l-L2. (A-F) Larvae in l-L2 were used to examine expression of Dll (A-F, blue), Lim1 (A-F, red), Ser (A’-C’, magenta), Dl (A’-C’, green), and *fng*-*lacZ* (D’-F’, white) in group 1 (A, D), group 2 (B, E) and group 3 (C, F) stages. Ser and Dl expressions are shown as maximum intensity projections. Optical sections along the yellow line are shown to the right of respective XY images. Quantitative expression analyses are shown in Fig 6F and 6G. Scale bars: 50μm.
(TIFF)

S8 Fig Patterns for Notch activation, *bantam*, and Ena during EAD A1 fold formation, and their effects on cell proliferation and apoptosis. (A-D) *Su(H)Gbe*-*lacZ* larvae were dissected to assesse N activity (lacZ, magenta), *bantam* (RNA *in situ*, blue) and Ena (white) expressions in the wing disc (A-B) and EAD (C-D). (A) In l-L3 wing disc, cells at the D/V boundary (high N activity, within dashed lines) show decreased *bantam* level. (B) Control scramble sequence showing non-specific signal in the D/V boundary (dashed lines). (C) In l-L2, N activity is elevated slightly in the presumptive A1 fold cells, where *bantam* and Ena are weak and ubiquitous. (D) In e-L3, cells in the A1 fold (arrows) show enhanced N activation, reduced *bantam* level, and increased Ena expression. (E) Percentage of mitotic cells (Phospho-Histone 3 over DAPI) in control, *N*^*DN*^, and *bantam*-overexpressing cells driven by *dpp*-*GAL4*. (F) Apoptotic cells (cleaved caspase 3, red) were similarly examined and quantified. The numbers of discs analyzed in control, *N*^*DN*^, and *bantam* overexpression were 11, 12, and 16 (proliferation), and 12, 11, and 12 (apoptosis) respectively. Mean values of proliferation/apoptosis, in control, *N*^*DN*^, and *bantam* were 0.55/0.19, 0.39/0.06, and 0.38/0.12, respectively. Scale bars: 50 μm, except in A-B: 25μm. * P ≤ 0.05 ** P ≤ 0.01 (ANOVA-Dunnett’s multiple comparisons).
(TIFF)

S1 Movie Apical areas in folded and non-folded cells during A1 fold formation. (AVI)

S2 Movie CALI on sqh-GFP combined with clonal tracking (RFP) experiment. RFP random clones (magenta) in sqh-GFP (green) were induced 24h prior to *ex vivo* EAD culture. Before CALI treatment, the EAD was carefully examined for the relative position between RFP clones and the A1 fold in xy (dashed line) and in xz (arrow) sections. Cells in yellow-boxed region were subjected to CALI treatment. In the presented case, RFP clones close to the CALI targeting region were mostly in the Dll field but not in the Lim1 field (see both xy and xz cross sections before CALI). After CALI treatment, two time-lapsed images were shown simultaneously. One for the RFP clones with Sqh-GFP, and the other for RFP alone with cell tracking (trajectories were color-coded according to time map, and shown in dragon tail mode). Spots indicated cells that were selected for trajectories analysis. Two peripodial RFP cells (arrows head, their positions at T_final_ were shown in Fig 5C”, 5F and 5G) appeared suddenly in the disc margin from post CALI time points 04:21:18 and 06:01:48, respectively. At time point post CALI 06:01:08, the A1 fold was reformed (see zoom-in image shown in single xy slice), and two RFP clones had crossed the A1 fold (dashed line). Once the A1 fold was reformed, there was no more cross-boundary observed for the RFP cells. Overall trajectories are shown after time-lapse images. RFP cells closed to the CALI targeting region (red spot) located in the Lim1 field at the end point (Fig 5C” and 5D). In this movie, two tracked cells near the CALI region crossed the A1 boundary, while five tracked cells, including one near the CALI region and four away from the CALI region, did not cross the A1 boundary. A total of three discs were analyzed, and five tracked cells that closed to the CALI targeting region have crossed the A1 boundary.
(MP4)

S3 Movie Clonal tracking (RFP) in Sqh-GFP without CALI. RFP clones without CALI treatment did not cross the A1 fold. In this movie, four cells were tracked and none crossed the A1 boundary. A total of six discs were analyzed and 13 cells were tracked (S6C Fig). None have crossed the A1 boundary during the imaging time for an average of 12 hours.
(AVI)

S1 Table Apical and basolateral domain height and volume in EAD cells. (DOCX)
