# Supplementary figures and images for: Notch-dependent epithelial fold determines boundary formation between developmental fields in the Drosophila antenna
Source: PLoS Genet. 2017 Jul 14;13(7):e1006898. doi: 10.1371/journal.pgen.1006898 (PMC5533456; doi:10.1371/journal.pgen.1006898)

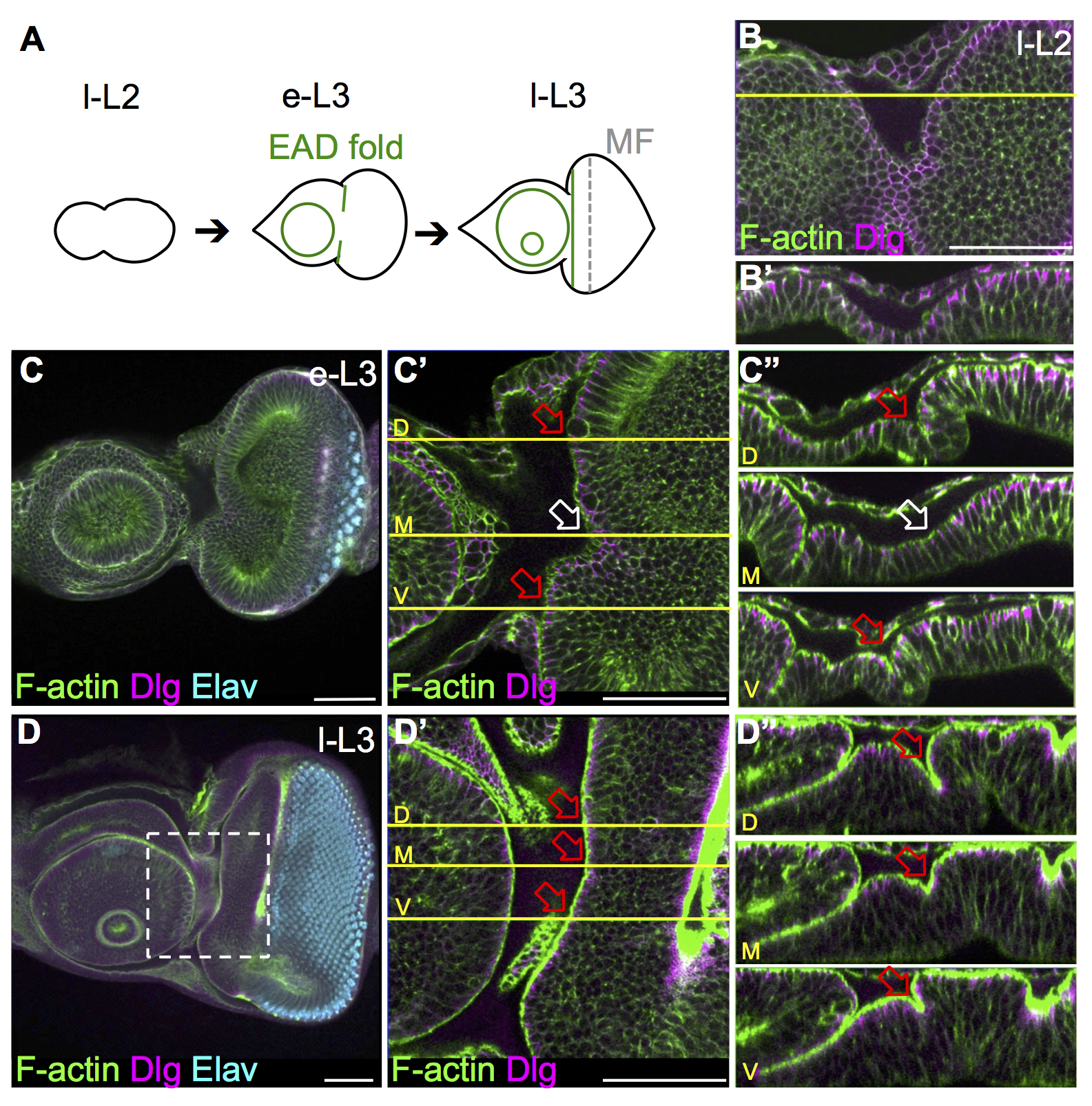

Supplement: S1 Fig — (A) The progressive formation of epithelial folds (green) in the EAD at the successive developmental stages is depicted. The morphogenetic furrow (MF) in eye disc is indicated by a grey dotted line. (B-D) EAD morphological changes are revealed by F-actin (green) and Dlg (magenta, basolateral domain) staining on w1118. (B-B’) During l-L2, the medial epithelial cells undergo a transition from a cuboidal to columnar shape, resulting in a concave morphology in the lateral view. (B’) Z-projection of optical sections at the yellow line in (B). (C-C”) In e-L3 EAD, the antennal field showed one completed ring of folding (the A1 fold) whereas in the eye field, epithelial folding only occurred in the lateral region (dorsal (D) and ventral (V) optical sections, red arrows) but not in the medial region (M, white arrow). (D-D”) In l-L3 EAD, the epithelial fold (red arrow) between the eye and antenna fields has formed completely. The Ar fold has formed in the antenna disc. Scale bars: 50μm (TIFF) [file pgen.1006898.s001.tiff]

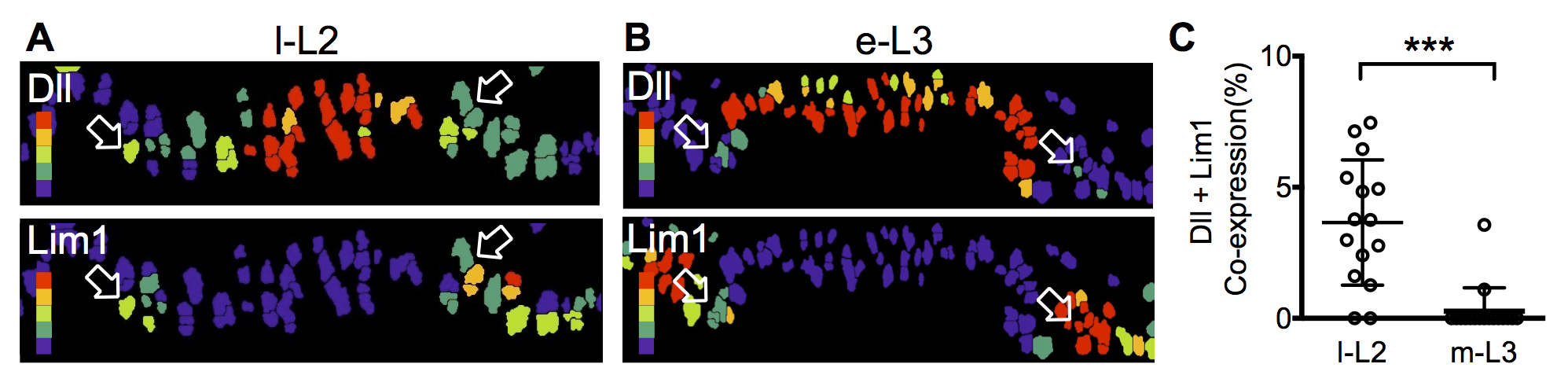

Supplement: S2 Fig — Cross-sections of Dll, Lim1, and DAPI staining were analyzed for quantitative expression. Nuclei counters were obtained from DAPI signal. (A-B) Average pixel intensities of Dll and Lim1 were grouped and color-coded (red to purple, high to low) from l-L2 (A) and e-L3 (B) EAD. Cells coded in purple were considered as not expressing either Dll or Lim1. Arrows pointing to cells show co-expression of Dll and Lim1. (C) Percentages of cells co-expressing Lim1 and Dll were quantitated. Mean ± stdev of co-expression in l-L2 and e-L3 were 3.65 ± 2.38% (N = 18) and 0.27 ± 0.89% (N = 20), respectively. *** P ≤ 0.001(two-tailed un-paired t test). (TIFF) [file pgen.1006898.s002.tiff]

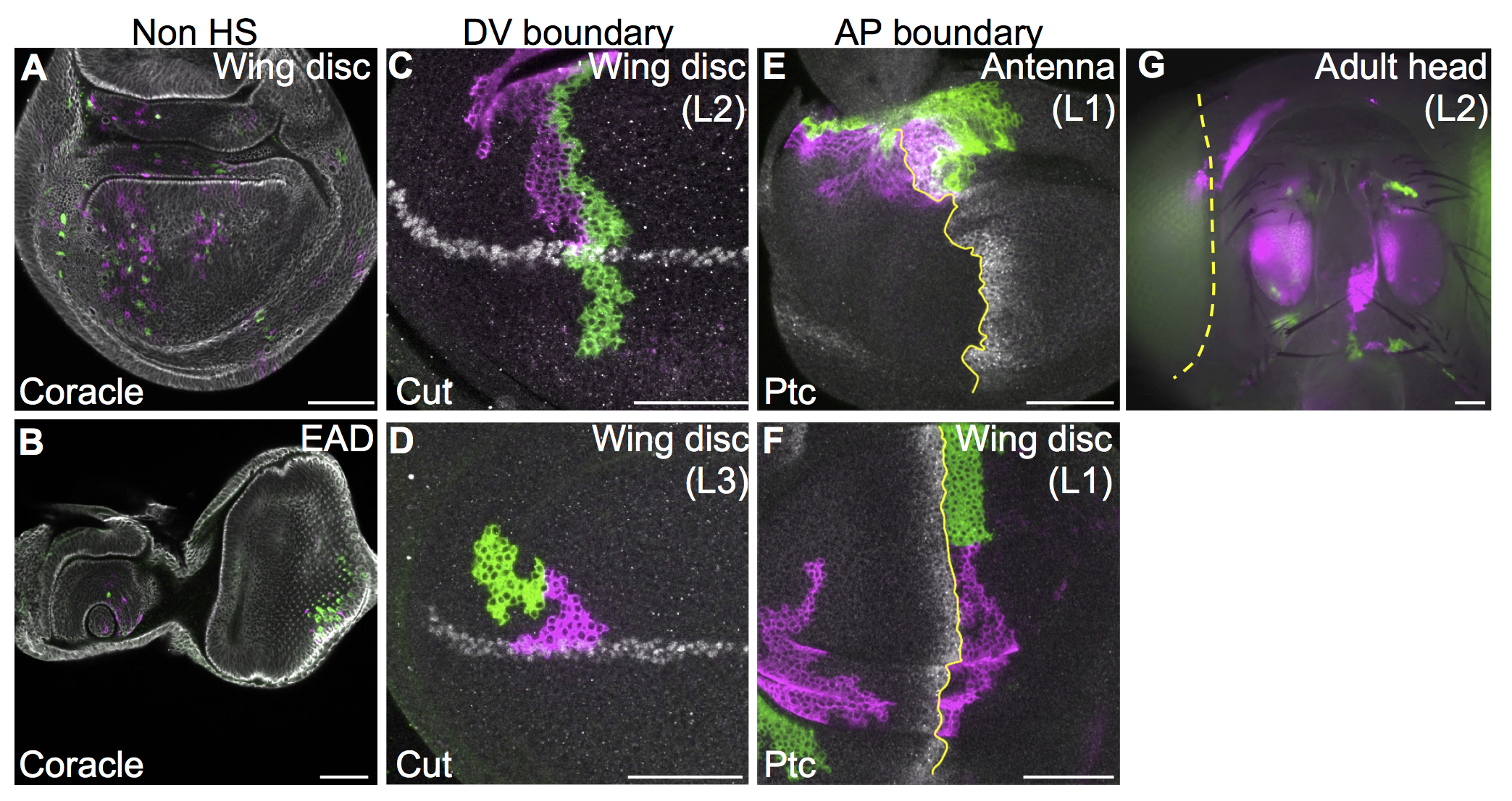

Supplement: S3 Fig — Sister clones are marked by GFP (green) and RFP (magenta), respectively. (A-B) 6% of discs (wing disc: 7/108, and EAD: 13/202) without heat-shock (Non-HS) showed non-specific, random GFP or RFP expressions. The non-specific signals are consistently weak, small (2–3 cells) and unpaired. (C-D) The D/V boundary (labeled by Cut expression, white) in the wing disc is not formed in L2 since clones induced at L2 cross the D/V boundary (C, 20/23). Instead, it is formed at early L3, since clones induced at early L3 do not cross the D/V border (D, 27/31). (E) Clones induced at L1 can cross the A/P boundary (marked by Ptc, white, and delineated by a yellow line) in the antennal disc. Clones induced at L2 can still cross the A/P boundary (marked by Ptc, 17/25), consistent with an earlier study showing that the A/P boundary in the antenna disc is not complete at 72h AEL [97]. (F) Clones induces at L1 are restricted by the A/P boundary in the wing disc (31/31). (G) TSM clones in adult fly head. Clones induced at the L2 crossed the boundary between head cuticle and compound eye (yellow line), indicating no lineage restriction between these two tissues at the L2 stage. Scale bars: 50μm. (TIFF) [file pgen.1006898.s003.tiff]

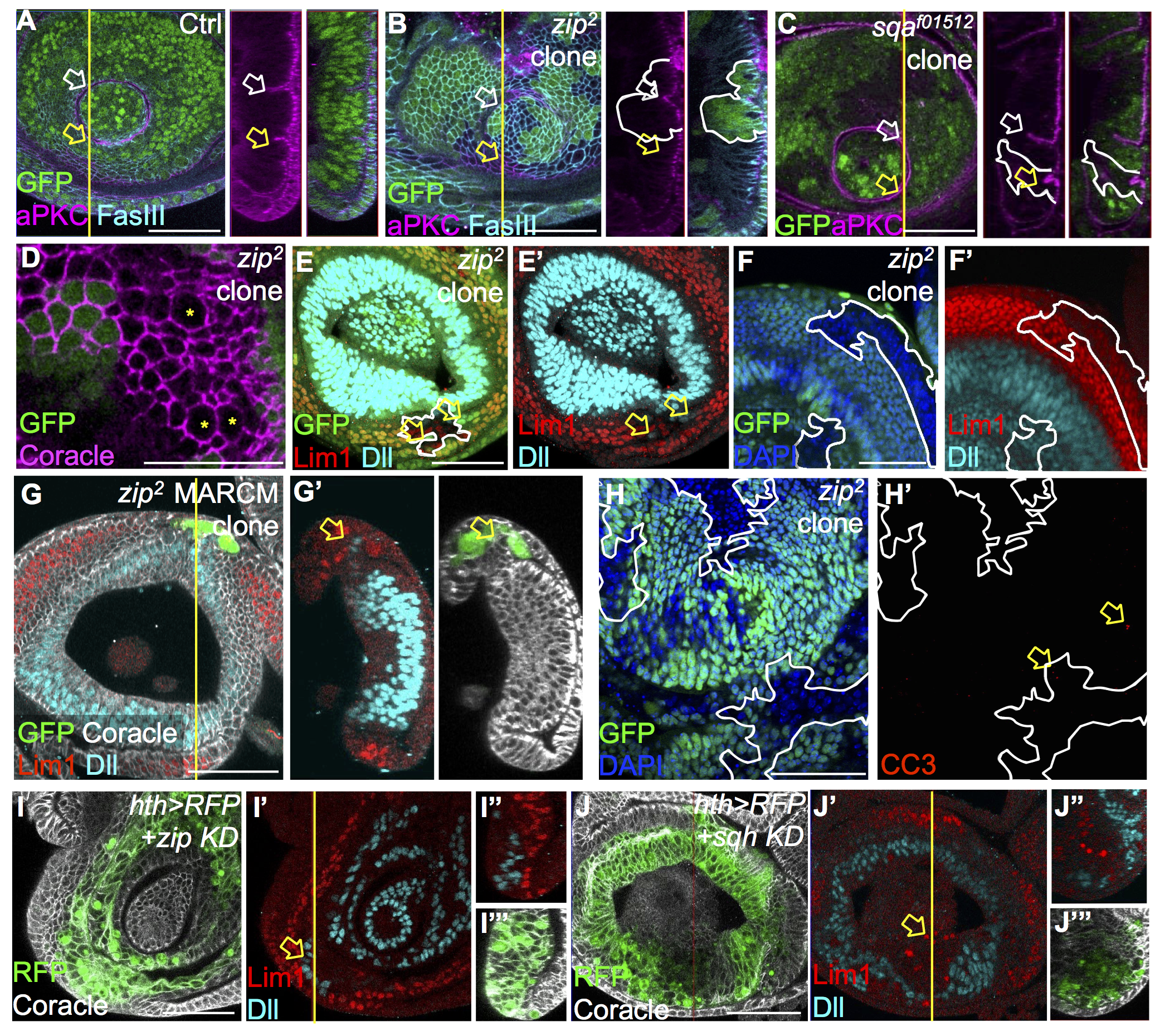

Supplement: S4 Fig — (A-C) Control (A), zip2 (B), and sqaf01512 (C) clones were analyzed for tissue morphology. Clones (no GFP, green) were induced in L1 and examined in l-L3. The optical section along the yellow line is shown on the right of each panel. In contrast to control, zip2 and sqaf01512 clones showed reduced or absent epithelial fold (compare white and yellow arrows in A, B, and C), but with normal apical-basal polarity (revealed by aPKC, magenta; FasIII, blue). (D) In zip2 clones (marked by absence of GFP, green), the mutant cells are enlarged. (E-E’) Some Dll cells are mislocalized (arrows) to the Lim1 domain, and can be seen at low frequency (4/23) outside of the zip2 mutant clone. (F) zip2 clones within a single field did not change the expression of Dll or Lim1. (G) In zip2 MARCM clones (mutant marked by GFP, green), a mixture of Dll and Lim1 (arrow) cells remained in the disc proper but were not sorted out for elimination. (H) Cleaved caspase 3-staining of zip2 clones. A few apoptotic cells (arrows) were detected. (I-J) Mixtures of Dll and Lim1 cells (arrow) following zip or sqh knockdown; mislocalized cells are maintained in the epithelial sheet (cross sections in I”-I”‘, J”-J’”). Scale bars: 50μm, except in D: 10μm. (TIFF) [file pgen.1006898.s004.tiff]

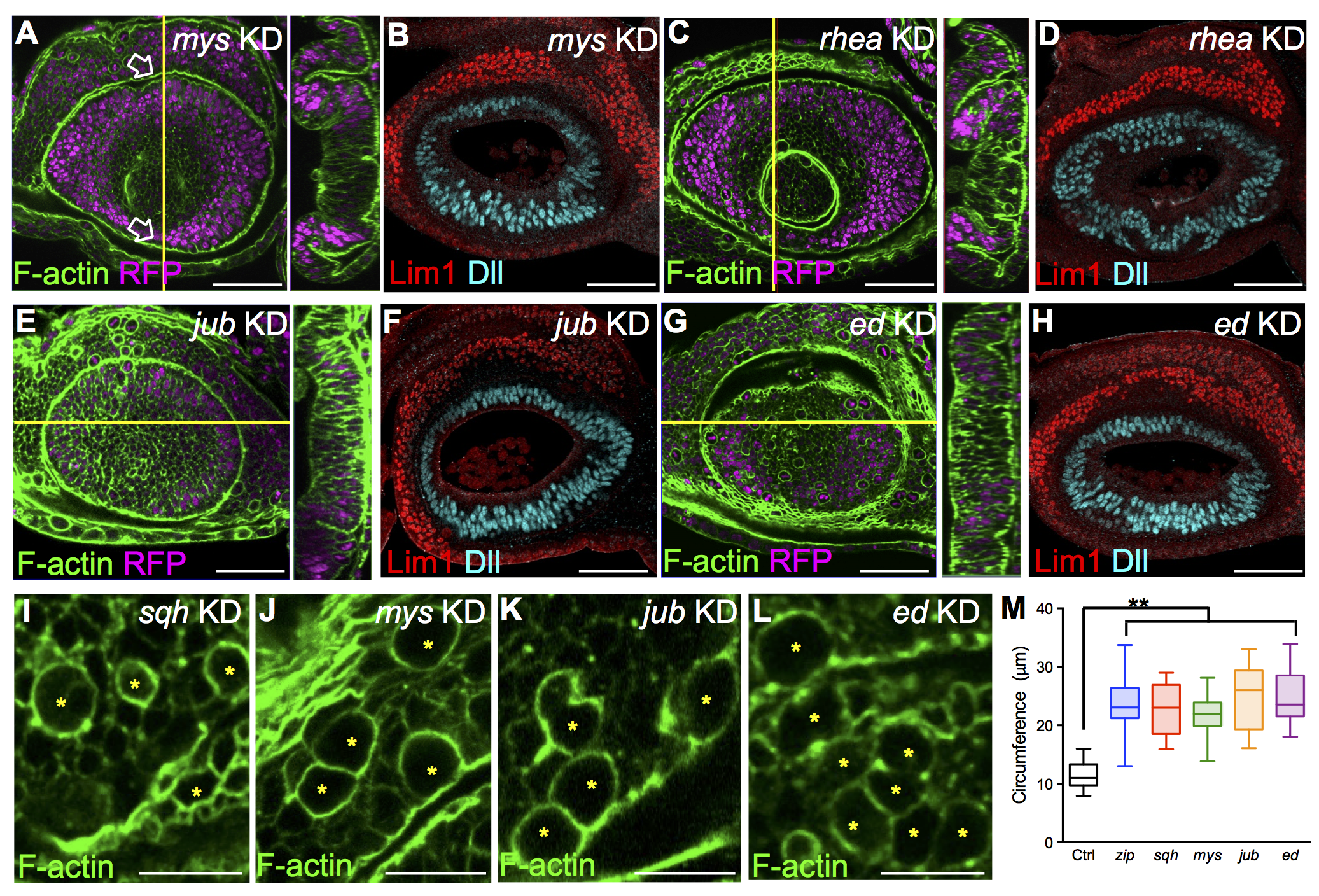

Supplement: S5 Fig — (A-H) Cell morphology (F-actin, green) and A1 fold (arrow in the Z-axis projection along the yellow line) were examined following knockdown of specific proteins in the proximal domain that encompass the A1 boundary, driven by hth-GAL4 (marked by RFP, magenta). (A-D) Knockdown of β-integrin (mys, A-B), or talin (rhea, C-D) causes cell enlargement, presumably due to a lack of basal focal adhesion, but did not affect the A1 fold. The Dll (blue) and Lim1 (red) domains remained sharply segregated (B and D). (E-H) Knockdown of jub (E-F) or ed (G-H) does not affect the A1 fold or Dll/Lim1 segregation. (I-L) Cell enlargement and/or delamination (indicated by stars) in sqh (I), mys (J), jub (K), and ed (L) knockdown mutants. (M) For each cell, serial focal planes were examined and the maximum circumference was selected for quantification. The average circumferences from single cells in different genotypes were compared. Ctrl. (mean ± stdev): 11.61 ± 2.36 (N = 21); zip KD: 23.37 ± 4.86 (N = 17); sqh KD: 22.94 ± 4.24 (N = 18); mys KD: 21.63 ± 3.45 (N = 21); jub KD: 24.9 ± 5.17 (N = 19); ed KD: 24.86 ± 4.61 (N = 20). Scale bars: 50μm, except in I-L: 10μm. (TIFF) [file pgen.1006898.s005.tiff]

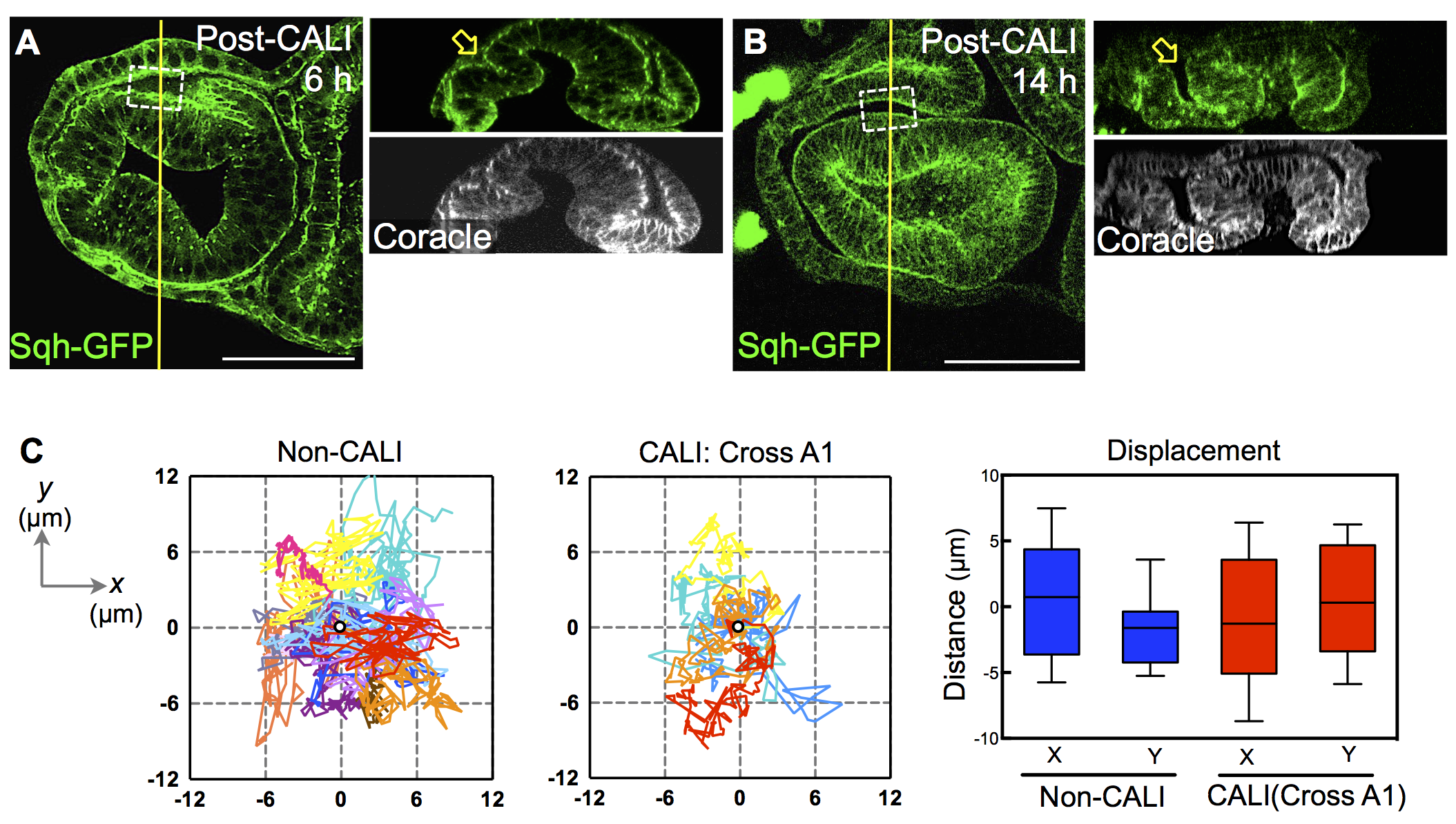

Supplement: S6 Fig — (A-B) Post-CALI (boxed region) EAD cultured for additional 6 (A) or 14 (B) hours were examined for the A1 fold (arrow in cross section) and gross morphology via Sqh-GFP (green) and Coracle (white) staining. (C) Overlay of cell trajectories in the CALI (for cells that crossed the A1 fold, N = 5) and non-CALI (N = 13) region. Displacements from T0 position (aligned in the center, for CALI = post CALI 0:00:00) are drawn. Each color line represents one cell. The average tracking time was 12 hours. The orientation and displacement in the x and y axis were not significantly different between the cells in the CALI and non-CALI (two-tailed un-paired t test). Scale bars: 50μm. (TIFF) [file pgen.1006898.s006.tiff]

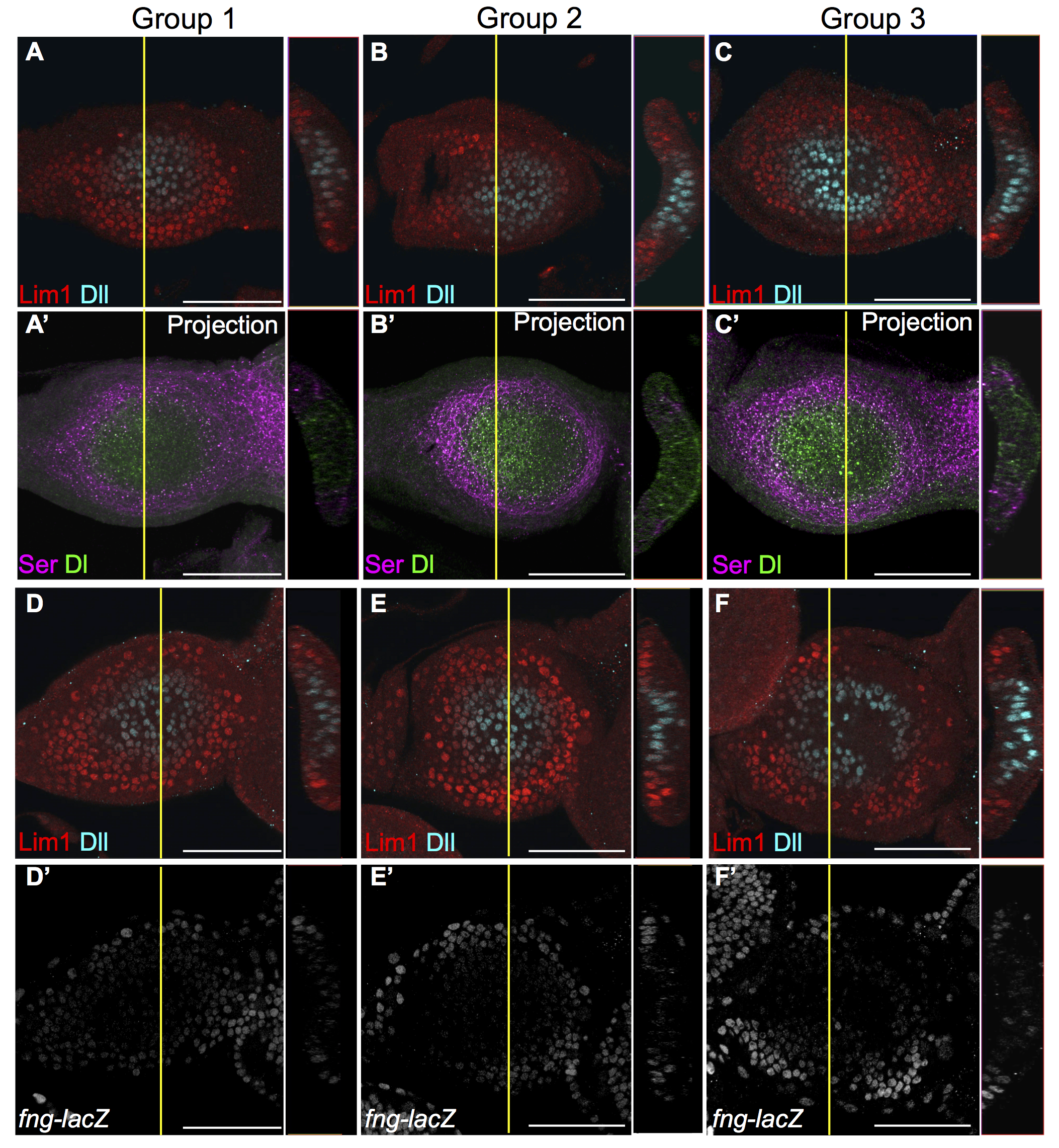

Supplement: S7 Fig — (A-F) Larvae in l-L2 were used to examine expression of Dll (A-F, blue), Lim1 (A-F, red), Ser (A’-C’, magenta), Dl (A’-C’, green), and fng-lacZ (D’-F’, white) in group 1 (A, D), group 2 (B, E) and group 3 (C, F) stages. Ser and Dl expressions are shown as maximum intensity projections. Optical sections along the yellow line are shown to the right of respective XY images. Quantitative expression analyses are shown in Fig 6F and 6G. Scale bars: 50μm. (TIFF) [file pgen.1006898.s007.tiff]

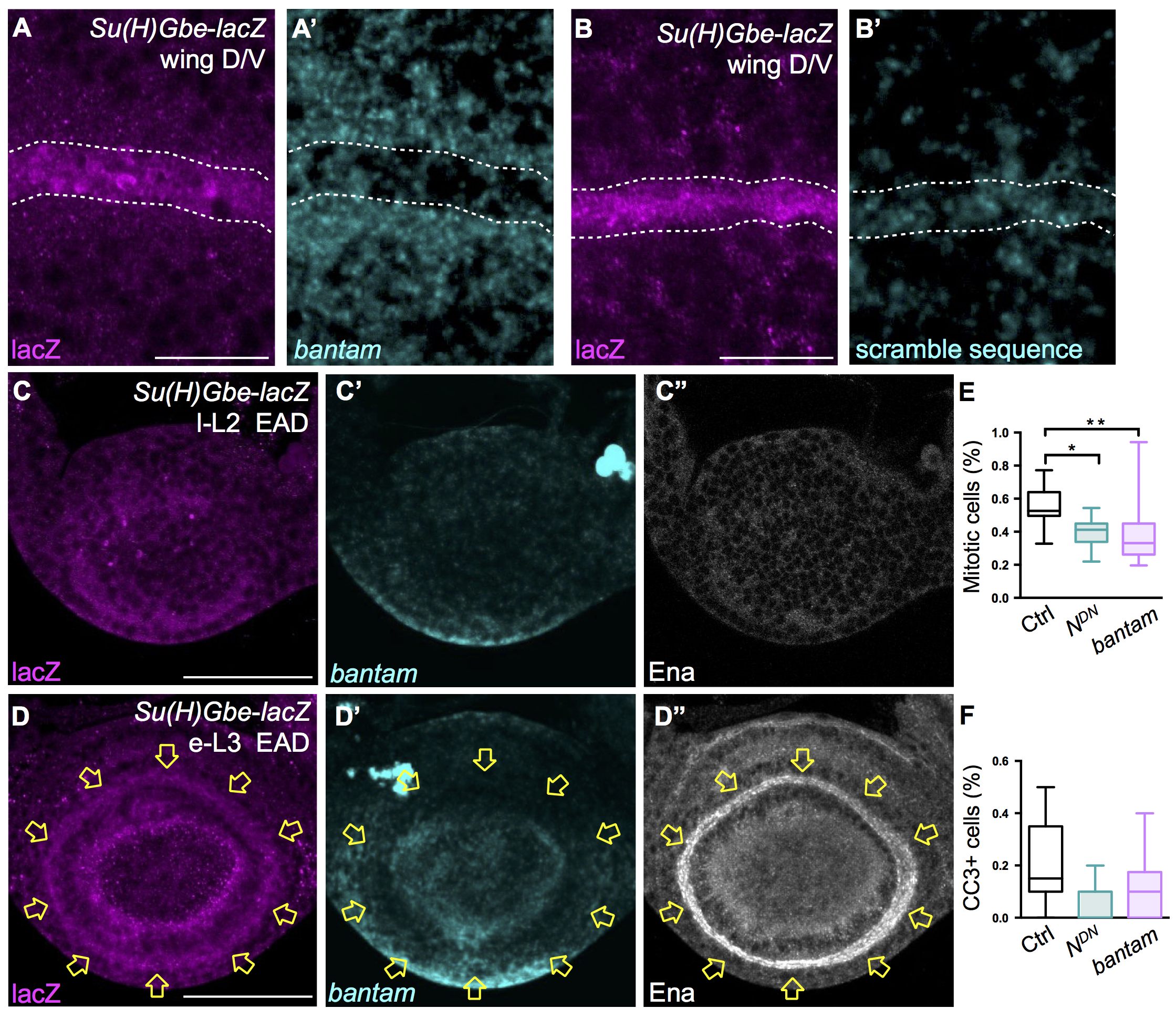

Supplement: S8 Fig — (A-D) Su(H)Gbe-lacZ larvae were dissected to assesse N activity (lacZ, magenta), bantam (RNA in situ, blue) and Ena (white) expressions in the wing disc (A-B) and EAD (C-D). (A) In l-L3 wing disc, cells at the D/V boundary (high N activity, within dashed lines) show decreased bantam level. (B) Control scramble sequence showing non-specific signal in the D/V boundary (dashed lines). (C) In l-L2, N activity is elevated slightly in the presumptive A1 fold cells, where bantam and Ena are weak and ubiquitous. (D) In e-L3, cells in the A1 fold (arrows) show enhanced N activation, reduced bantam level, and increased Ena expression. (E) Percentage of mitotic cells (Phospho-Histone 3 over DAPI) in control, NDN, and bantam-overexpressing cells driven by dpp-GAL4. (F) Apoptotic cells (cleaved caspase 3, red) were similarly examined and quantified. The numbers of discs analyzed in control, NDN, and bantam overexpression were 11, 12, and 16 (proliferation), and 12, 11, and 12 (apoptosis) respectively. Mean values of proliferation/apoptosis, in control, NDN, and bantam were 0.55/0.19, 0.39/0.06, and 0.38/0.12, respectively. Scale bars: 50 μm, except in A-B: 25μm. * P ≤ 0.05 ** P ≤ 0.01 (ANOVA-Dunnett’s multiple comparisons). (TIFF) [file pgen.1006898.s008.tiff]
