# Supplementary material for: Notch-dependent epithelial fold determines boundary formation between developmental fields in the Drosophila antenna
Source: PLoS Genet. 2017 Jul 14;13(7):e1006898. doi: 10.1371/journal.pgen.1006898 (PMC5533456; doi:10.1371/journal.pgen.1006898)
Supplement: S1 Table — (DOCX) [file pgen.1006898.s012.docx]

## S1 Table. Apical and basolateral domain height and volume in EAD cells

|  | | Height (μm) | | Volume (μm^3^) | |
| --- | --- | --- | --- | --- | --- |
|  |  | **Apical** | **Basolateral** | **Apical** | **Basolateral** |
| l-L2 | | 1.03 ± 0.24 | 17.25 ± 1.16 | 6.19 ± 2.02 | 157.26 ± 26.88 |
| e-L3 | **non-folded** | 0.93 ± 0.14 | 18.89 ± 1.71 | 5.45 ± 2.06 | 177.83 ± 28.36 |
|  | **folded** | 0.89 ± 0.13 | 10.12 ± 1.28 | 1.42 ± 0.34 | 90.225 ± 20.94 |
|  | **peri-folded1** | 0.96 ± 0.21 | 12.44 ± 2.30 | 2.94 ± 1.03 | 114.40 ± 22.99 |
|  | **peri-folded2** | 0.89 ± 0.13 | 13.40 ± 2.59 | 4.80 ± 1.80 | 124.84 ± 30.14 |

Mean ± stdev are shown. Number in each group=8.
